# A Scale-Corrected Comparison of Linkage Disequilibrium Levels between Genic and Non-Genic Regions

**DOI:** 10.1371/journal.pone.0141216

**Published:** 2015-10-30

**Authors:** Swetlana Berger, Martin Schlather, Gustavo de los Campos, Steffen Weigend, Rudolf Preisinger, Malena Erbe, Henner Simianer

**Affiliations:** 1 Animal Breeding and Genetics Group, Department of Animal Sciences, Georg-August-University, Goettingen, Germany; 2 School of Business Informatics and Mathematics, University of Mannheim, Mannheim, Germany; 3 Department of Epidemiology and Biostatistics, Michigan State University, East Lansing, Michigan, United States of America; 4 Institut of Farm Animal Genetics, Friedrich-Loeffler Institut, Neustadt-Mariensee, Germany; 5 Lohmann Tierzucht GmbH, Cuxhaven, Germany; Nanjing Forestry University, CHINA

## Abstract

The understanding of non-random association between loci, termed linkage disequilibrium (LD), plays a central role in genomic research. Since causal mutations are generally not included in genomic marker data, LD between those and available markers is essential for capturing the effects of causal loci on localizing genes responsible for traits. Thus, the interpretation of association studies requires a detailed knowledge of LD patterns. It is well known that most LD measures depend on minor allele frequencies (MAF) of the considered loci and the magnitude of LD is influenced by the physical distances between loci. In the present study, a procedure to compare the LD structure between genomic regions comprising several markers each is suggested. The approach accounts for different scaling factors, namely the distribution of MAF, the distribution of pair-wise differences in MAF, and the physical extent of compared regions, reflected by the distribution of pair-wise physical distances. In the first step, genomic regions are matched based on similarity in these scaling factors. In the second step, chromosome- and genome-wide significance tests for differences in medians of LD measures in each pair are performed. The proposed framework was applied to test the hypothesis that the average LD is different in genic and non-genic regions. This was tested with a genome-wide approach with data sets for humans (*Homo sapiens*), a highly selected chicken line (*Gallus gallus domesticus*) and the model plant *Arabidopsis thaliana*. In all three data sets we found a significantly higher level of LD in genic regions compared to non-genic regions. About 31% more LD was detected genome-wide in genic compared to non-genic regions in *Arabidopsis thaliana*, followed by 13.6% in human and 6% chicken. Chromosome-wide comparison discovered significant differences on all 5 chromosomes in *Arabidopsis thaliana* and on one third of the human and of the chicken chromosomes.

## Introduction

In genomic studies, associations between traits of interest and genomic polymorphisms are sought. In most whole genome marker data sets, the causal variants are generally not included but the effects of quantitative loci are reflected by markers that are in linkage disequilibrium (LD) with the causal loci (e.g. [1]). For this reason, LD has become particularly instrumental in mapping genes that cause diseases [2, 3, 4]. LD patterns also reflect the demographic development and demographic processes like migration and admixture and can be used to infer respective parameters (e.g. [2, 5, 6]). Awareness of LD patterns in the genome is thereby essential for correctly interpreting results from Genome-Wide Association Studies (GWAS). Rare variants will only be captured if they are in high LD with observable markers, which is only possible if the MAF of the causal variant and the marker are of similar magnitude [7, 8]. In populations with a limited effective population size, such as breeding populations, high LD extends over long physical distances. In such cases, methods utilizing LD mapping allow for more efficient usage of low density single nucleotide polymorphism (SNP) chips already available for genomic selection [7, 9, 10].

Large-scale data from high density SNP chips provide fine scale resolution LD maps for many species [11, 12, 13] and can be used to analyze the genome-wide LD structure. A wide range of scientific insights or groundbreaking findings based on LD patterns has been gained in human genetics [14, 15, 16] and in population genetics [10, 12, 17, 18].

Factors like mutation, recombination, selection, or genetic drift have a strong impact on the development and dynamics of the non-random association between loci. Influence of MAF on LD is disturbing the genetic analysis. Both, the decay of the non-random association between the SNPs with growing physical distance (e.g. [10]) and the dependency of most measures of LD on minor allele frequency (MAF) are well known [19]. Hence, different remedies have been suggested. For instance, Garner and Slatkin [20] used a subset of markers selected on the basis of allele frequencies for association studies, other methods (e.g. [21, 22]) are based on various kinds of standardization to minimize the influence of MAF on LD measures. For example, the dependency of the disequilibrium coefficient *D* on MAF is reduced by standardizing with its maximum, but the resulting measure reaches its maximum value only if less than four gametes are observed. Other less MAF dependent methods need haplotype data (e.g. index of association, homozygosity of haplotypes [23], normalized entropy difference [24] or are of parametric nature (e.g. Kullback-Leibler distance [25])).

Deeper insight into the LD structure of the genome, especially in genic regions, will also help to identify relationships between traits of interest and genetic variants, to improve the understanding of biological processes and also may increase the accuracy of estimating genomic effects. Many studies investigating the association between the loci compare the LD level in different populations (e.g. [15, 26]), but only a few studies compared the magnitude of the LD in genic versus non-genic regions. McVean et al. [2] indicated higher recombination rates outside of genic regions in the human genome, suggesting a higher rate of LD within genes. Smith et al. [6] reported the proportion of genes in different quartiles of LD, while Kim et al. [13] presented the proportion of genic markers in LD hotspots. Eberle et al. [27] evaluated the decay of LD in genic and inter-genic regions by assessing the number of perfectly correlated SNPs. To avoid the bias due to differences in MAF, the authors used only a small subset of available SNPs for the analysis that had identical MAF. Eberle et al. [27] observed a higher fraction of perfectly correlated SNPs in genic regions compared to intergenic regions, however these observations are valid only for the specific subset of SNPs and cannot be automatically generalized to other not pre-selected sets of SNPs. So far, a general procedure for comparing LD levels between different genomic regions that uses the comprehensive information and accounts for various potential sources of bias is missing. A key challenge when comparing LD patterns between different regions in the genome is to eliminate the impact of MAF on LD. An additional difficulty is that the density of markers varies across chromosomes and different SNP chips [28] and is different for genic and non-genic regions, which may lead to a structural bias on LD measures.

To overcome the MAF driven limitations of LD measures and the bias caused by genome topology variations we propose a general framework for comparison of LD magnitude in different genomic regions by applying the following methodology, which is structurally similar to matched pairs design used in clinical studies (e.g. [29]): (a) identification of pairs of regions with most similar characteristics (MAFs, pairwise MAF differences, pairwise physical distances), (b) determination of the LD levels for each matched pair of regions, and (c) application of the Wilcoxon signed rank test to the paired LD measures at chromosome-wide or genome-wide level. Best matching regions are identified by comparing the empirical cumulative distribution functions (ECDF) of the considered variables in both regions. To assess the extent of linkage disequilibrium we used the squared correlation (*r* ^2^) derived from phased haplotypes, a widely used statistic describing the association between two loci [19]. We rescaled *r* ^2^ using the bounds given by VanLiere and Rosenberg [30] to achieve a less MAF dependent measure of LD. The suggested approach was applied to test the hypothesis that the level of LD is higher in genic than in non-genic regions. We applied our approach to three real data sets: for humans (*Homo sapiens*), a highly selected chicken line (*Gallus gallus domesticus*) and the model plant *Arabidopsis thaliana*.

## Materials and Methods

### Statistical methods

In a diploid organism, there are four possible combinations of alleles at two bi-allelic loci (locus 1 with major allele *A* or minor allele *a* and locus 2 with major allele *B* or minor allele *b*) called gametes *AB*, *Ab*, *aB* or *ab*. For ease of notation, only the frequencies of minor alleles *p*_1_ at locus 1 and *p*_2_ at locus 2 were used, since the major allele frequencies can be expressed as 1-*p*_1_ and 1-*p*_2_, respectively. The coefficient of gametic (phase) disequilibrium D, also called disequilibrium coefficient, measures the differences between the observed frequency *p*_12_ of gamete *ab* and its expectation under independence, yielding *D* = *p*_12_ − *p*_1_ *p*_2_.

The disequilibrium coefficient *D* builds a basis for several measures of allelic association. Pearson’s correlation coefficient *r* for a 2x2 contingency table representing gametic frequencies can be rewritten as $r=\frac{D}{\sqrt{p_{1}(1-p_{1})p_{2}(1-p_{2})}}$. Note that the absolute value, but not the sign of r is insensitive to an arbitrary labeling of alleles, and thus the Pearson’s squared correlation coefficient *r* ^2^ is an appropriate measure of LD which was first used by Hill and Robertson [31] to describe the extent of LD in finite populations. The authors also recognized that the range (and other characteristics) of this statistic depend on the allele frequencies, which was intensively considered in later studies (e.g. [32, 33, 34]). VanLiere and Rosenberg [30] suggested $r_{s}^{2}=r^{2}/r_{\text{max}}^{2}$, where $r_{\text{max}}^{2}$ is the maximum possible value of *r* ^2^ given the respective MAFs at the two loci considered. For our studies, squared correlations *r* ^2^ as well as $r_{S}^{2}$ were used to determine the amount of LD in compared genomic regions.

### Accounting for scale effects

We consider the general problem of testing whether the LD structure differs between certain genomic regions, such as genic vs. non-genic regions, each region being represented by a number of sets of SNPs (a set may e.g. represent all SNPs in a gene). The basic idea of our approach is, similar to the matched pairs design [29], for a given reference set of SNPs to find a best matching control set (a set may e.g. represent SNPs in a non-genic chromosomal region) with the same number of SNPs that is most similar in all characteristics known to affect the LD measures. For each pair of matching sets, LD measures were calculated and averaged. Finally statistical tests were performed across all pairs of sets to verify whether the median differences are significantly different.

#### Identifying best matching sets

**Fig 1:**
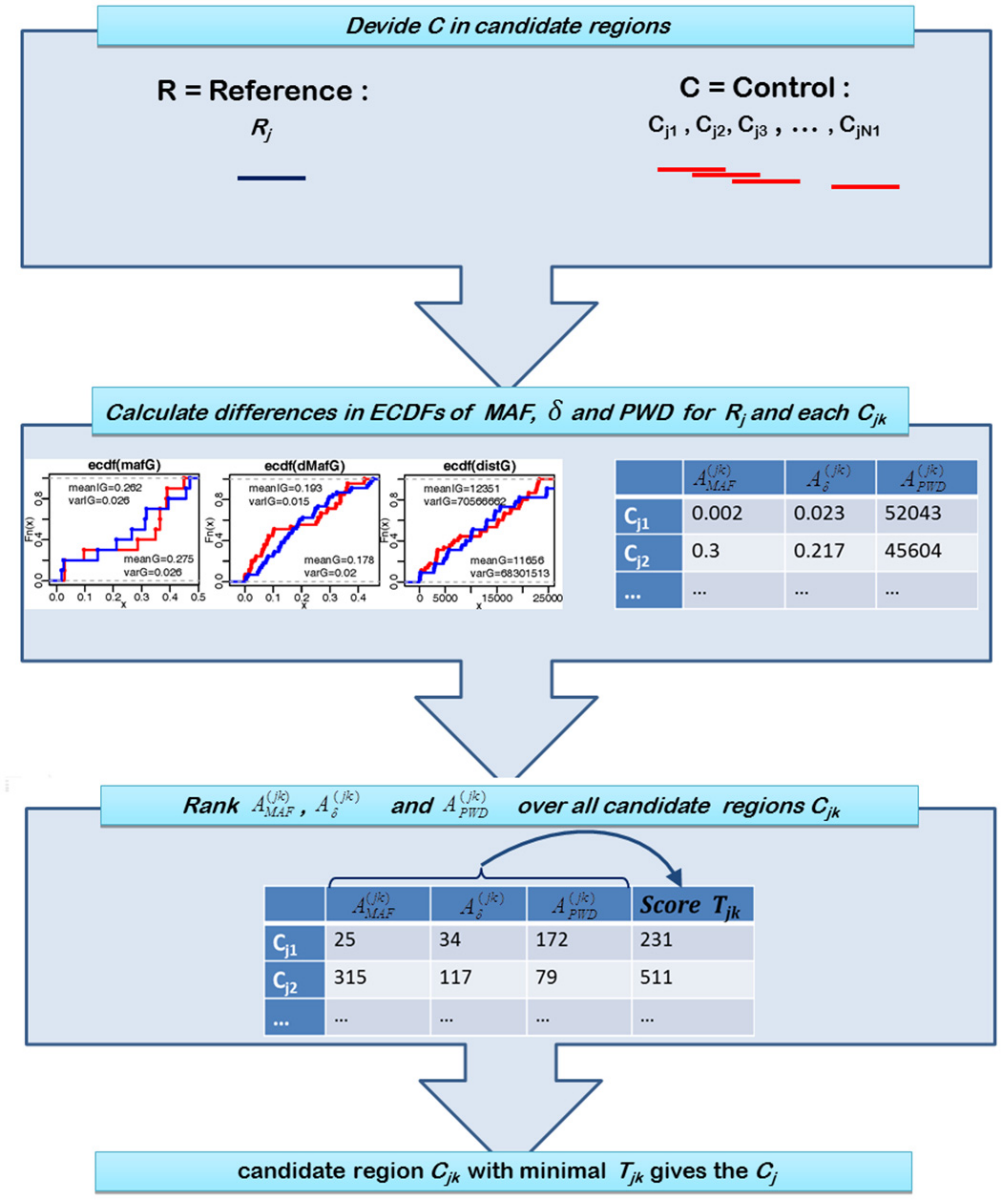
Work flow for identifying best matching sets.

We denoted a reference set (for example a gene) consisting of *m*_*j*_ SNPs as *R*_*j*_, and the best matching set of markers with the most similar characteristics on the chosen scales as the control set *C*_*j*_ (for example subset of markers from a non-genic region). We used MAFs, pairwise differences between the MAFs (*δ*), and pairwise physical distances (PWD) as most relevant characteristics to identify similarity between genomic regions. To identify this best matching control set *C*_*j*_, the control region was divided into *N*_*j*_ candidate subsets $C_{j1},\ldots ,C_{jk},\ldots C_{jN_{j}}$ by sliding windows of size *m*_*j*_ SNPs (see Fig 1). The larger the reference set, the smaller the number of candidate subsets *N*_*j*_. To achieve stability of estimates, we excluded any reference sets with less than 10 SNPs or less than 50 candidate subsets *C*_*jk*_ from further analysis, since a sufficient similarity between *R*_*j*_ and the best matching *C*_*j*_ might not be assured in these cases.

For each reference set *R*_*j*_ and candidate subset *C*_*jk*_, the empirical cumulative distribution functions of MAFs, pairwise differences between the MAFs, and pairwise physical distances, were calculated separately. For each of the variables the area (A) between the ECDF curves for the reference set *R*_*j*_ and candidate subset *C*_*jk*_, (also called Wasserstein metric [35], [36]) was determined, which was denoted as $A_{MAF}^{\left(jk\right)}$, $A_{\delta }^{\left(jk\right)}$, and $A_{PWD}^{\left(jk\right)}$, respectively (an example is given in S1 Fig). For selecting a control set *C*_*jk*_ which is most similar in all characteristics, we ranked firstly all $A_{MAF}^{\left(jk\right)}$, $A_{\delta }^{\left(jk\right)}$ and $A_{PWD}^{\left(jk\right)}$ over *k* = 1,…,*N*_*j*_ in each characteristic separately. Finally an overall score $T_{j1},\ldots ,T_{jk},\ldots ,T_{jN_{j}}$ was built by summing up those three ranks for each *C*_*jk*_ to a total score *T*_*jk*_ The candidate subset *C*_*jk*_ with the lowest overall score was linked as matching control set *C*_*j*_ to the reference set *R*_*j*_.

This approach was used to ensure that differences in LD are not caused by the differences in the size of regions (measured in number of SNPs or as accumulated physical distances) or by differences in the distribution of allele frequencies, but are only caused by the affiliation to a genic vs. non-genic region.

#### Determining the differences in LD level and statistical significance testing

For all pairs of SNPs within each *R*_*j*_ and each *C*_*j*_ we calculated *r* ^2^ and determined their medians ${\hat{m}}_{R_{j}}$ and ${\hat{m}}_{C_{j}}$, respectively. The Wilcoxon signed rank test was then applied to compare the LD level in both regions and to test the null hypothesis that the median difference between pairs of $m_{R_{j}}$ and $m_{C_{j}}$ is equal to zero against the alternative hypothesis that this median difference is not equal to zero (two-sided testing). The comparisons are performed chromosome-wise as well as at the genome-wide level. Similar calculations were performed for $r_{S}^{2}$. In all tests we used a 5% significance level.

### Data

The LD structure in genic and non-genic regions was investigated using data from three different species: *Arabidopsis thaliana*, *Homo sapiens* and *Gallus gallus domesticus* (a summary for all three data sets is given in Table 1)

**Table 1:** Summary of data sets used across all species.

Species	Sample size	No. of chromosomes studied	No. of		No. of SNPs		
			genes annotated	genic regions studied	total	genic	non-genic
***A*. *thaliana***	199	5	33,323	3,721	215,947	135,768	80,179
***H*. *sapiens***	5,961	22	54,849	7,180	684,990	391,576	293,414
***G*. *g*. *domesticus***	673	26	17,108	3,033	277,522	146,963	130,559

#### Arabidopsis thaliana

We used an *A*. *thaliana* data set published by Atwell et al. [37]. Data consisted of 199 unique accessions, fully homozygous inbred lines, which had been genotyped using the Affymetrix 250 K SNP-tiling array (AtSNPtile1), and was downloaded from http://archive.gramene.org/db/diversity/diversity_view?db_name=diversity_arabidopsis&action=view&object=div_experiment&id=5. We removed 14 SNPs with missing genotype rate greater than or equal to 0.01 and 170 SNPs with MAF less than 0.01. All individuals passed quality control and the missing genotypes rate per individual was less than 0.0001 leaving 215,947 SNPs for downstream analysis.

Gene annotations were drawn from http://plants.ensembl.org version ‘Ensembl plant genes 21’ [38], based on the current Arabidopsis Information Resource (TAIR) 2009-10-TAIR 10 assembly (http://www.arabidopsis.org). Only genes annotated from chromosome 1 to 5 were used, resulting in a total of 33,323 genes. All overlapping genes were merged to single gene regions. We selected for the analysis those genes that had at least 10 SNPs; in all 3,721 gene regions were considered.

#### Human (*Homo sapiens)*

The genotypes used for the data analysis in humans were taken from the Gene-Environment Association Studies (GENEVA [39], www.genevastudy.org). We used a subset of GENEVA consisting of data from the Nurses’ Health Study and the Health Professionals’ Follow-up Study. Samples had been genotyped using the Affymetrix Genome-Wide Human SNP Array 6.0 with about 780 K SNPs. The data set contained genotypic records of 5,961 individuals.

We removed all markers with a proportion of missing genotypes per SNP greater than or equal to 0.01 and all individuals with a proportion of missing genotypes per individual greater than or equal to 0.05. Furthermore, on the basis of available pedigree information, we also removed all nominally related individuals and individuals with a Hispanic genomic background so that only unrelated individuals of Caucasian origin remained in the data set. We also set a lower threshold of 0.01 for MAF. After quality control of genomic data sample size of 5,827 individuals genotyped at 684,990 SNPs loci remained.

We used gene annotations from http://ensembl.org version ‘Ensembl genes 74’ [40]. Only genes annotated from chromosome 1 to 22 were used, which resulted in a total of 54,849 genes that comprised 20,364 coding genes, 20,070 non-coding genes and 14,415 pseudogenes. After merging overlapping genes and dropping out all genic regions with less than 10 SNPs, 7,180 genic regions were retained for further analysis.

#### Chicken (*Gallus gallus domesticus*)

We used 673 individuals of a highly selected White Leghorn chicken line from a Synbreed (www.synbreed.tum.de) data set. Samples had been genotyped using the Affymetrix Axiom^®^ Genome-Wide Chicken Genotyping Array [41] with about 600 K SNPs. None of the individuals showed a missing genotype rate greater than or equal to 0.05, while SNPs with missing genotype rate greater than or equal to 0.01 and MAF less than0.01 were removed. After quality control a sample of size 673 individuals and 277,522 SNPs remained. We used gene annotations from http://ensembl.org version ‘Ensembl genes 74’ [40]. 17,108 genes annotated from chromosome 1 to 28 (except chromosomes 16 and 24), were used. The SNP coverage of chromosomes 16, 24 and all small chromosomes greater than 28 was not sufficient for the analysis. Upon merging the overlapping genes and removing genic regions with less than 10 SNPs, we were left with 3,033 genic regions for the analysis.

Density of markers, expressed as the number of SNPs per physical distance unit, varied across species: in *A*. *thaliana* the SNP density was around 3.0–3.6 SNPs per kilo base pair (SNPs/kbp), while in *H*. *sapiens* 0.20–0.36 SNPs/kbp were available. In *G*. *g*. *domesticus* the density of markers varied across chromosomes: for chromosomes 1 to 8 the marker density was very similar to the one in the human data set, while on chromosomes 9 to 28 the density of SNPs was about 0.4–1.0 SNPs/kbp. For all data sets, additional information about the distribution of allele frequencies, marker densities in genic and non-genic regions is available in S1, S2, S3, S4, S5 and S6 Figs.

### Data analysis

We used the framework described above to compare LD levels in genic and non-genic regions in the human, chicken, and Arabidopsis genome. In addition, as a control, the comparison between two similar non-genic regions was performed. Imputing of missing genotypes as well as haplotype-phasing was performed using the BEAGLE software (version 3.3.2) [42].

Before starting the analysis, some data editing was necessary: overlapping genes were observed in all species, meaning that a gene was either lying completely within another gene or two genes overlapped partially. All overlapping genes were merged to one ‘genic region‘, since overlapping genes are inherited together with high probability [43, 44].

All markers in-between these genic regions were assigned to non-genic regions. For each genic region *G* we selected one most similar non-genic region *IG*, using the procedure described above. In an independent procedure we chose another *IG* set, termed *IG’*, as a control, which is most similar to the *IG* but does not overlap with *IG*. In general, we searched for the best matching *IG* and *IG’* on the same chromosome as *G*. Due to the small size of chromosomes in *G*. *g*. *domesticus* from chromosome 6 onwards, we joined these chromosomes to a single chromosomal region and searched for the best matching *IG* and *IG’* in this chromosomal region. The control comparison of best matching *IG* and *IG’* pairs will assure that discovered differences in *G/IG* pairs are not caused by the selection procedure, thus we expect no differences in LD level in I*G/IG’* pairs.

We applied a two-sided Wilcoxon signed rank test with the null hypotheses *H*_0_:Δ_**G/IG**_ = 0 or *H*_0_:Δ_**IG/IG'**_ = 0 versus alternatives *H*_1_:Δ_**G/IG**_ ≠ 0 and *H*_1_:Δ_**G/IG'**_ ≠ 0, where Δ_**G/IG**_ refers to median differences in *G/IG* pairs and Δ_**IG/IG'**_ described median differences in *IG/IG’* pairs. Tests are performed using chromosome- or genome-wide sets of *G*, *IG* and *IG’*.

Depending on the region of the genome we looked at, we expected genic and non-genic regions to differ not only in the extent of LD, but also in the haplotype frequencies. We used the haplotype diversity *H* to describe the variation in haplotype frequencies in a region, which is defined as [45]:

$$H=\frac{m}{m-1}\left(1-\overset{2^{m}}{\underset{i=1}{\sum }}f_{i}^{2}\right)\in \left[{}^{}0{,}^{}1^{}\right],$$

where *m* is the number of SNPs in the considered region (*G*, *IG* or *IG’*) and *f*_*i*_ is the (relative) haplotype frequency of the *i* ^*th*^ haplotype out of the 2^*m*^ possible haplotypes. The relative haplotype frequency $f_{i}=\frac{n_{i}}{N}$ describes the proportion of the *i* ^*th*^ haplotype in all existing haplotypes in the considered genomic region.

We applied a two-sided Wilcoxon signed rank test with the null hypotheses *H*_0_ : *δ*_**G/IG**_ = 0 and *H*_0_ : *δ*_**G/IG'**_ = 0 versus alternatives *H*_1_ : *δ*_**G/IG**_ ≠ 0 and *H*_1_ : *δ*_**G/IG'**_ ≠ 0 for the haplotype diversities in *G/IG* and *IG/IG’* comparisons. The parameters *δ*_**G/IG**_ and *δ*_**G/IG'**_ refer to median differences in haplotype diversity in *G/IG* and *IG/IG’* pairs, respectively.

The identification procedure for *G/IG* and *IG/IG’* pairs as well as all statistical analyses were implemented in R [46]. The smoothing curves of pair-wise measures, based on natural cubic splines, was prepared using R-package ggplot2 [47].

## Results

**Fig 2:**
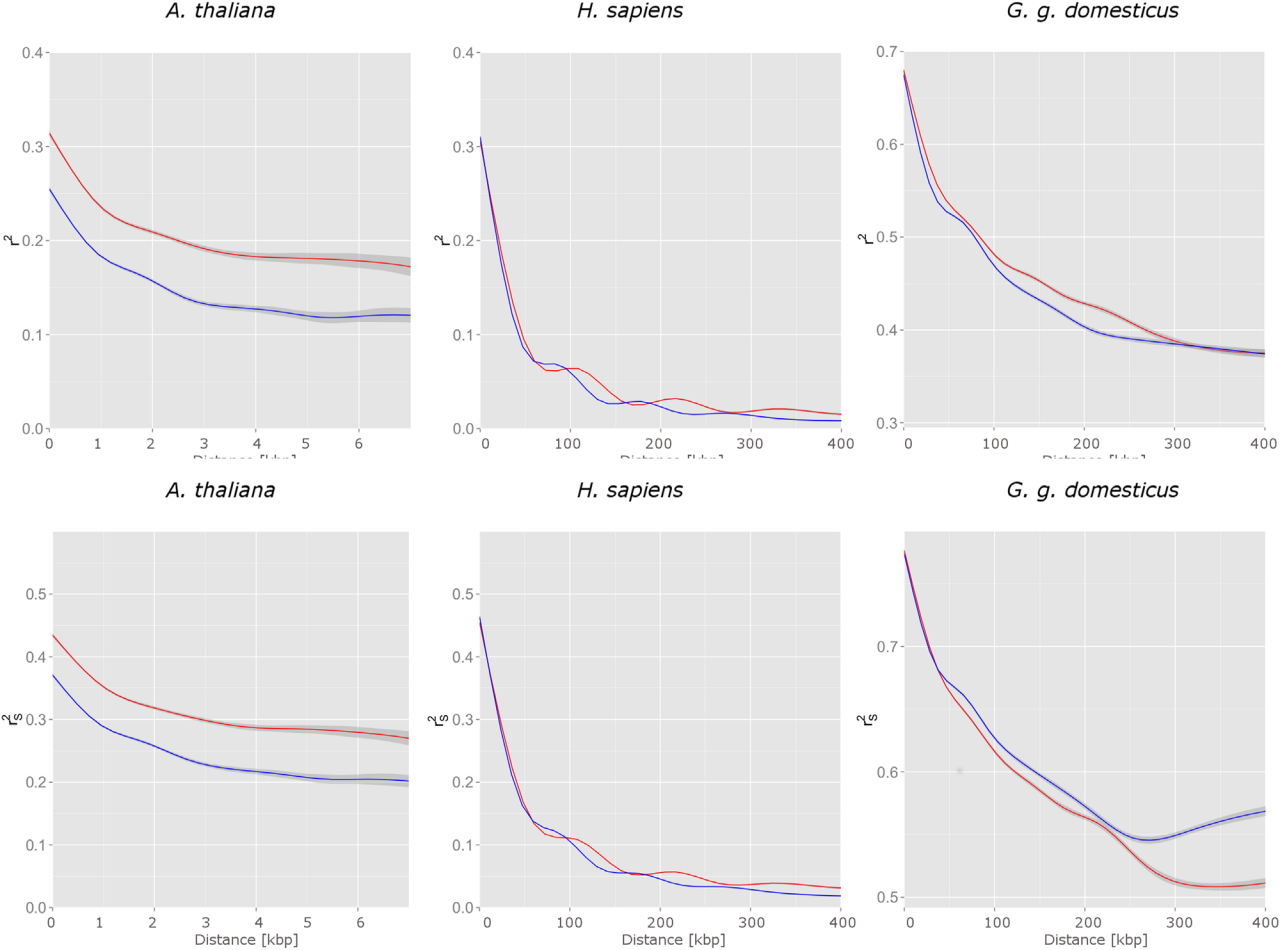
Smoothed curves of squared correlation coefficients *r* ^2^ (upper panel) and $r_{S}^{2}$ (lower panel), calculated for SNP pairs in genic regions (red lines) versus matching non-genic regions (blue lines) with confidence regions (shaded gray) in *A*. *thaliana*, *H*. *sapiens* and *G*. *g*. *domesticus*, plotted against the physical distance in kilo base pairs.

A first comparison of the amount of the LD in genic and non-genic regions was done based on smoothed curves of *r* ^2^ against the physical distance. Here we considered SNPs comprising 99% of all SNP pairs, excluding the upper 1% of SNP pairs with large distances. At distances >7 kbp in *A*. *thaliana* and distances >400 kbp in *H*. *sapiens* and *G*. *g*. *domesticus*, only a few pairs of SNPs existed (see S7 Fig) and therefore were excluded from the analysis. A kernel smoothing of pair-wise *r* ^2^ and $r_{S}^{2}$ measures is displayed in Fig 2. The amount of LD at very short distances in *A*. *thaliana* was comparable to that observed in *H*. *sapiens*, but the decay was much faster in *A*. *thaliana*: SNPs located more than 7 kbp apart have *r* ^2^ measures around 0.12 in non-genic regions and around 0.17 in genic regions, while in *H*. *sapiens r* ^2^ at this distance still is about 0.25 in both genic and non-genic regions. As expected, in the commercial chicken line we observed a high amount of LD in general, spanning over wide ranges. Regardless of the absolute levels of *r* ^2^, higher levels of LD in genic regions in contrast to non-genic regions were detected across all three species, most clearly in *A*. *thaliana*.

**Fig 3:**
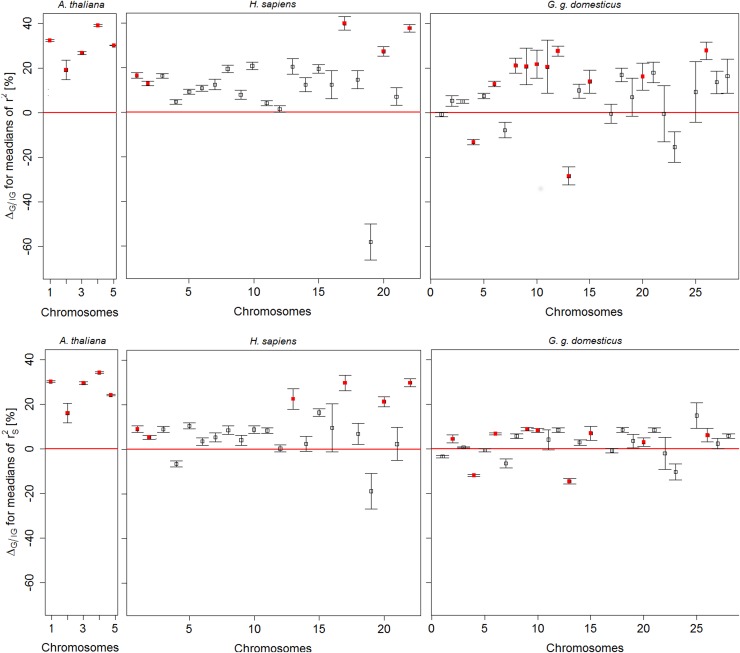
Comparison of genic (*G*) versus non-genic (*IG*) regions across chromosomes in *A*. *thaliana*, *H*. *sapiens and G*. *g*. *domesticus*. Chromosome-wise averaged percentage differences Δ_**G/IG**_ ± *se* between medians of *r* ^2^ in *G* and medians in *IG* (upper panel) and chromosome-wise averaged differences Δ_**G/IG**_ ± *se* between $r_{S}^{2}$ in *G* and in *IG* (lower panel), where *se* refer to standard errors of averages. Red filled symbols indicate significant differences in *G/IG* comparison.

The much higher average level of LD in the highly selected White Leghorn chicken population compared to the other species is reflected by an asymmetric distribution of pair-wise *r* ^2^: the center of mass was shifted to the smaller values in *H*. *sapiens* and *A*. *thaliana*, while in *G*. *g*. *domesticus* center of mass was located in the area with high values (see S8 Fig). Thus we chose the median as an appropriate summary statistic to describe LD in explored genic and non-genic regions and to quantify observed differences. The significance tests for chromosome-wise *G/IG* differences (*LD*_*G*_ − *LD*_*IG*_) in medians of *r* ^2^ and of $r_{S}^{2}$ yielded coherent results in most cases. Fig 3 shows the averaged percentage differences Δ_**G/IG**_ = (*LD*_*G*_ − *LD*_*IG*_)/*LD*_*G*_·100% with corresponding standard errors, which are plotted against the chromosome numbers for all species (for more details see S1, S2, S3, S4, S5, S6, S7, S8 and S9 Tables).

In *G*. *g*. *domesticus* significant median differences in $r_{S}^{2}$ at 7 chromosomes (Fig 3, lower panel) were positive and thus confirmed the assumption of higher LD level in genic compared to non-genic regions. This seems to be in conflict with the observation that over long distances the smoothed curve of pair-wise $r_{S}^{2}$ for non-genic regions is higher than that for genic regions (Fig 2, lower panel). This might be due to the fact that an increased level of LD in genic regions is predominantly found in shorter chromosomes, while in some of the large chromosomes (1, 4) LD in genic regions is less than that in non-genic regions (Fig 3).

When fitting a linear regression within species, the coefficient of determination between averages per chromosome calculated for *r* ^2^ and chromosome-wide averages calculated for $r_{S}^{2}$ was high for all species: 0.75 in *H*. *sapiens*, 0.78 in *G*. *g*. *domesticus* and 0.79 in *A*. *thaliana*. So, decisions of Wilcoxon signed rank test based on the LD measure *r* ^2^ corresponded to the test decisions made for differences in a MAF independent measure $r_{S}^{2}$. This consistency in test results has led to the conclusion that our framework was efficient in adjusting for spatial and for MAF influences.

In case of genome-wide comparison of medians of *r* ^2^ about 31% more LD was detected in genic regions than in non-genic regions in *A*. *thaliana*, followed by 13.6% in *H*. *sapiens* and 6% in *G*. *g*. *domesticus*. The comparisons of Δ_**G/IG'**_ between matching non-genic regions *IG* and *IG’* yielded no significant differences for *r* ^2^ but for $r_{S}^{2}$ a significant difference was found for one chromosome in *A*. *thaliana* and *G*. *g*. *domesticus*, respectively, which is in the expected range under the null hypothesis (S1, S2, S3, S4, S5, S6, S7, S8 and S9 Tables). The outcomes of chromosome-wise and genome-wide comparisons are summarized in Table 2.

**Table 2:** Number of chromosomes with significantly (p-value <0.05) increased LD level in the comparison of genic with matching non-genic regions (Δ_**G/IG**_), number of chromosomes with significantly different LD levels for matching non-genic regions (Δ_**G/IG'**_), and the genome wide average difference in LD between genic and matching non genic regions in per cent (Δ_**G/IG**_ [%]) for the two LD measures *r* ^2^ and $r_{S}^{2}$. Asterisks indicate the level of significance for the genome-wide differences.

Species	Chromosomes studied					Genome-wide	
	Total	Δ_**G/IG**_		Δ_**G/IG**_'		Δ_**G/IG**_[%]	
		*r* ^2^	$r_{S}^{2}$	*r* ^2^	$r_{S}^{2}$	*r* ^2^	$r_{S}^{2}$
***A*. *thaliana***	5	5	5	0	1	31.2***	27.7***
***H*. *sapiens***	22	5	5	0	0	13.6*	8.0**
***G*. *g*. *domesticus***	26	10	9	0	1	6.0**	0.5

*: p-value <0.05

**: p-value <0.01

***: p-value <0.001

We expected a higher LD in genic regions compared to non-genic regions and performed 53 chromosome-wide significance tests in total (Fig 3), 18 chromosomes (34%) showed a significantly higher LD in genic regions. In two chromosomes (chromosome 4 and 13 in chicken) significantly higher LD in non-genic regions was observed. This corresponds to 3,8% of all comparisons and is below the 5% significance level. Thus the unexpected results for chromosomes 4 and 13 might be the false positive test outcomes obtained just by chance.

The Wilcoxon signed rank test, applied chromosome-wise, detected significant differences between genic and non-genic regions on all 5 chromosomes of *A*. *thaliana*, on about 1/4 of the human chromosomes and on about 40 per cent of the chicken chromosomes.

**Fig 4:**
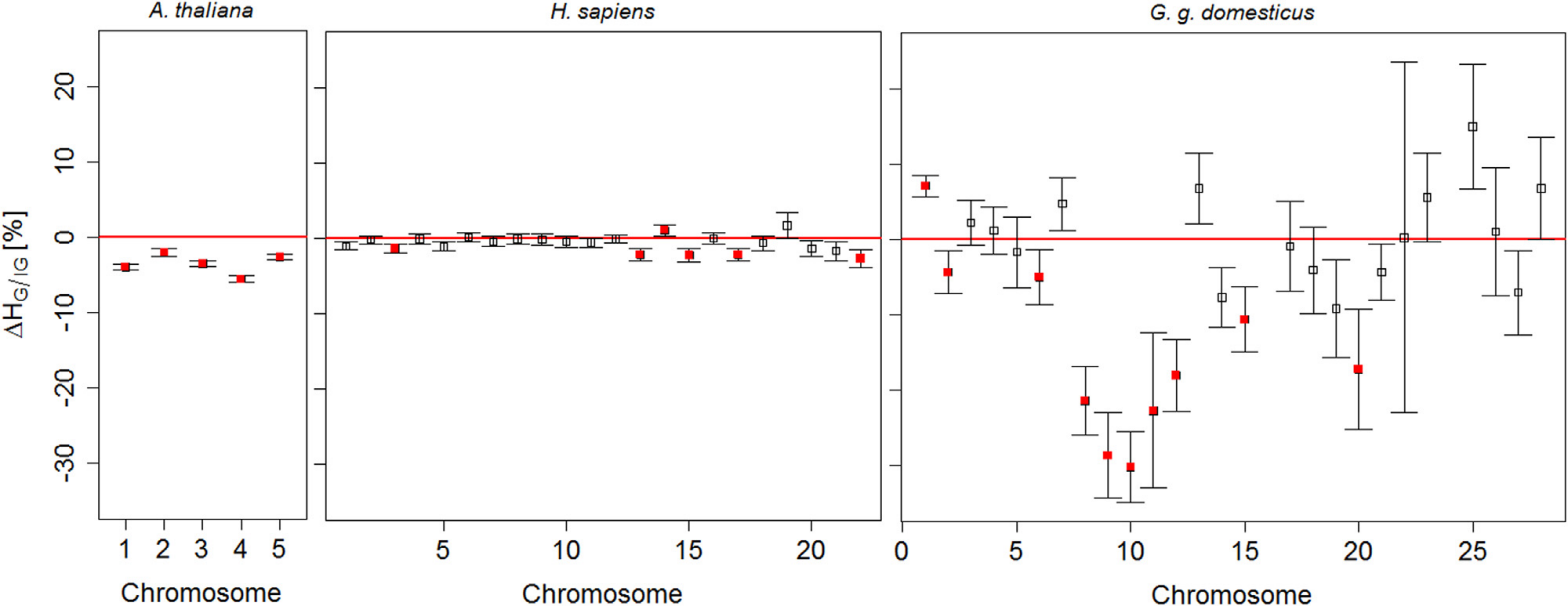
Chromosome-wise differences in haplotype diversity in *G/IG* comparisons, across species. Chromosome-wise haplotype diversity percentage differences Δ*H*_*G*/*IG*_ ± *se* plotted against the chromosome number, where *se* refers to standard errors of averages. Red filled symbols indicate significant (p-value <0.05) differences in *G/IG* comparison.

In Fig 4 chromosome-wise percentage differences in haplotype diversities Δ*H*_*G*/*IG*_ = (*H*_*G*_ − *H*_*IG*_)/*H*_*G*_·100% for the three species are presented.

The haplotype diversity in *A*. *thaliana* and *H*. *sapiens* were both relatively high, at a comparable level: chromosome-wide averages ranged between 0.85 and 0.89 in genic regions, accompanied by significantly lower haplotype diversity in *G* compared to *IG* (see S9 Fig and S10, S11 and S12 Tables). In *A*. *thaliana* we observed Δ*H*_*G*/*IG*_ = −3.5% less diversity in haplotypes at the genome-wide level, while the loss of haplotype diversity in G varied between -2% and -5% at the chromosome level. In *H*. *sapiens*, a small significant loss Δ*H*_G/IC_ = −0.7% was observed at the genome-wide level, whereas significant Δ*H*_G/IC_ varied between -0.7% and -2.6% at the chromosome level. In *G*. *g*. *domesticus*, haplotype diversity of −2.9% at the genome-wide level was significant, albeit smaller than that in *A*. *thaliana*, whereas the chromosome-wide averages in genic regions ranged between 0.40 and 0.61 and the significant Δ*H*_G/IC_ between −4.3% and −23.2% at the chromosome level was the largest of all three species.

## Discussion

Apart from the proportion of protein-coding DNA in the genome, the major question is whether the changes over generations are differently occurring in different genomic regions. We introduced a general comparison framework, which copes with difficulties arising while performing comparison of LD levels between different genomic regions, such as the impact of the extent of compared regions on the genome (spatial bias) and the impact of allele frequencies on LD (MAF caused bias). The retrieved knowledge about variation in genomic regions of interests could be used, for example, to estimate a measure for likelihood of fitness consequences of involved populations proposed by Gulko et al. [48].

### Impact of location of a region: genic versus non-genic regions

The results obtained for *A*. *thaliana* were in contrast to those obtained by Kim et al. [13], who suggested that LD hot spots in arabidopsis are situated preferentially outside genic regions. On a genome-wide level, significantly more LD in genic regions was observed in all three species and thus the observation by Eberle et al. [27] for the human genome was confirmed and quantified. The LD levels in genic regions at very short physical distances are similar in *A*. *thaliana* and *H*. *sapiens* with *r* ^2^ being about 0.3 on average (see Fig 2). In *A*. *thaliana* a clear gap between LD amount in genic and non-genic regions is seen while in *H*. *sapiens* almost no G/IG difference is recorded up to a distance of about 50 kilo base pairs, while in maize, which is in contrast to *A*. *thaliana* an outcrossing plant, or in self-pollinating barley a comparable decay of LD (up to 3 kbp) was observed by Caldwell et al. [49].

LD spans are so short and genic regions are more conserved in *A*. *thaliana* compared to humans presumably is due to the fact that *A*. *thaliana* is an ubiquitous plant and the sample used in our studies reflects a very large effective population size (N_e_) that may explain the rapid decay of LD. Contemporary estimates of N_e_ of *A*. *thaliana*, based on sequence data of 80 strains from a wide Eurasian region indicated N_e_ to lie between 250,000 and 300,000 [50]. The LD level observed in *G*. *g*. *domesticus* is twice as high as the LD level in *H*. *sapiens* and LD decays much slower than in humans. This higher LD level is observed in *G*. *g*. *domesticus* over all distances. The white layer data used originate from a commercial line, which has been intensively selected for egg laying in a closed nucleus breeding scheme. Thus the degree of relatedness among the individuals in the studied sample is relatively high: average pedigree based relatedness was 0.255±0.07 and the average inbreeding coefficient was 0.10±0.025. The magnitude of relatedness in the population has a strong impact on the effective population size, which is very low in commercial lines of chicken [49, 50]. For pair-wise distances ≤ 25 kbp, Qanbari at al. [51] reported values of *r* ^2^ between 0.60 and 0.74 in four different layer lines, which is concordant with the magnitude of LD detected in our study. Also the decay of LD observed in the white layer data set (*r* ^2^ ≈ 0.37 for pairs of SNPs in about 400 kbp distance) was consistent with results from previous studies (*r* ^2^ = 0.35 for pairs of SNPs in about 200–500 kbp distance [51, 52]). Layer breeding schemes use a small number of highly selected male individuals in each generation.

A similar monopolization of reproductive function by one or few individuals is also given in eusocial insects (like e.g. ants) causing reduced effective population size and a high degree of conservation in coding genomic regions [53].

Many statistical methods have been developed in the last decade to utilize high-throughput sequencing data for estimating population parameters (e.g. [51, 52]), among them a maximum-likelihood estimator of recombination rates based on LD patterns [54, 55]. Thus, stronger association observed between markers in genic regions than in non-genic regions might go along with a higher recombination rate in non-genic regions. Accordingly, a lower diversity of haplotypes is expected in genic regions compared to non-genic regions. Indeed significantly less diversity of haplotypes in genic regions was noticed for all species, which confirms our results obtained for LD.

Genic regions in general appear to be more conserved than non-genic regions (e.g. [27, 56,57]). Higher haplotype diversity in non-genic regions may be explained by the fact that recombination in these regions may affect biological cycles or pathways to a lesser extent; thus most haplotypes resulting from recombination will be neutral with respect to fitness and will not be under selection. In contrast, recombination in genic regions may affect the biological function of the respective haplotype and consequently such haplotypes with reduced fitness will be less frequently found among the progeny, resulting in a reduced haplotype diversity in genic regions. Regions with low recombination were found to contain highly conserved genes with essential cellular functions (e.g. [58]). Furthermore, hitchhiking and background selection might generate a strong link between genetic diversity and recombination rate [59, 60, 57]. Thus, the intensive anthropogenic selection in white layers may explain the pronounced differences between haplotype diversity in genic and non-genic regions in the white layer data.

### Impact of chromosome size or size of region on LD magnitude

The suggested approach accounting for spatial and structural differences in genomic regions when comparing genic and non-genic regions provides new insights into the dependency of LD levels on the size of chromosomes or regions. Assuming that the number of recombination events per chromosome is approximately equal, differences in recombination rates on chromosomes of different physical length are supposed [61, 6, 54] with a slower decay of LD in the larger chromosomes. In contrast to the findings of Smith et al. [6] and Uimari et al. [62] for the human genome and Hillier et al. [63] and Groenen et al. [64] for the chicken genome, we do not observe weaker LD in the smaller chromosomes and stronger LD in the large chromosomes (see S10 Fig and S13 Table). Even though the chromosome-wise averaged medians scattered more in *G*. *g*. *domesticus*, there was no clear association between the size of chromosomes and the level of LD. Considering the size of genic and non-genic regions across chromosomes, a weak but significant negative association between the size and the LD of a region was detected in all species. For instance, in *G*. *g*. *domesticus* larger regions showed a slightly lower *r* ^2^ (the slope of a fitted linear regression ≈ −0.002) and also slightly lower $r_{S}^{2}$ (the slope of a fitted linear regression ≈ −0.001, see S11 Fig). This size bias is expected since physically large genic regions have more pairs of physically distant SNPs, which in turn have a lower LD (see Fig 2). There was no significant size bias for the differences in medians of *r* ^2^ and of $r_{S}^{2}$ since we corrected for the effect of the length of the region through comparing with a region of similar size. This is exemplarily visualized for *G*. *g*. *domesticus* in S12 Fig.

Across all species the extent of LD measured in genic or non-genic regions did not depend on the size of the chromosome (see S13 Table). Discrepancies between our results and results reported by Smith et al [6] and Uimari et al. [62] may have resulted either from the lower marker density, lower SNP call rates and smaller sample sizes in these older studies or due to bias caused by spatial differences or different distribution of allele frequencies.

### Conclusions

Our study has shown that across the three considered species, the average level of LD is systematically higher in genic regions than in non-genic regions, confirming and quantifying the more qualitative result in the human genome of Eberle et al. [27] for a wider range of species. This observed difference is not affected by other factors which might systematically differ between genic and non-genic regions, such as minor allele frequencies or SNP densities, since such differences were removed by comparing candidate sets with best matching counterparts. With this approach, it was also possible to exactly quantify the relative excess of LD on a chromosome-wise or genome-wide level. It was shown that the amount of excess LD in genic regions differs between species (with *A*. *thaliana > H*. *sapiens > G*. *g*. *domesticus*) and varies substantially between the chromosomes within the considered species. These observations found for the widely used LD-measure *r* ^2^ in tendency were confirmed with the standardized LD-measure $r_{S}^{2}$ and with haplotype diversity. Based on our findings we suggest that the excess of LD in genic region is a general phenomenon resulting from evolutionary forces, since the patterns of genetic polymorphisms reflects evolutionary processes like recombination, genetic drift and selection.

The suggested approach can be varied by replacing the squared correlation *r* ^2^ by any other LD measure (e.g. D’ [65], homozygosity of haplotypes [23], normalized entropy difference [24] or Kullback-Leibler distance [25]), by accounting for more or different scaling factors or by varying the similarity score by using different weighting of those factors. The comparative assessment of the LD level in genic and non-genic regions might be used as a starting point for a more differentiated analysis of the LD structure in the genome. In our studies we applied just two categories of genomic regions: genic and non-genic regions, where genic regions were defined in accordance with annotations of known genes in Ensembl gene databases. This way of proceeding is coherent to the classification of genic regions used by Eberle et al. [27] and provides us better comparability to their results. A promising area for improvement of our current approach is the extension of considered genetic regions by a stratification in e. g exons, introns, 5k upstream or downstream regions, 5’ and 3’ UTRs etc. Such analyses might require higher marker densities (up to sequence level) and considerably enlarged sample sizes, though. An especially interesting subject for further research is the contribution of purifying and positive selection across breeding populations to differences in level of LD between coding and non-coding regions of the genes. The framework described here enables comparison of LD structure in arbitrary species and any genomic regions of interests.

## Supporting Information

S1 FigArea between the Empirical Cumulative Density Functions.ECDFs for reference set (red) and for a candidate subset (blue), the $A_{MAF}^{\left(jk\right)}$ (left), $A_{\delta }^{\left(jk\right)}$(center), and $A_{PWD}^{\left(jk\right)}$(right) are marked in grey.(TIFF)

S2 Fig SNP-density for chromosomes 1 to 5 in *A*. *thaliana*. Red bars stand for density of SNPs in genic regions, blue bars stand for SNP-density in non-genic regions.
(TIFF)

S3 Fig Distribution of minor allele frequencies in *A*. *thaliana* across the whole genome, in genic and in non-genic regions, respectively. (TIFF)

S4 Fig SNP-density for chromosomes 1 to 22 in *H*. *sapiens*. Red bars stand for density of SNPs in genic regions, blue bars stand for SNP-density in non-genic regions.
(TIFF)

S5 Fig Distribution of minor allele frequencies in *H*. *sapiens* across the whole genome, in genic and -non-genic regions, respectively. (TIFF)

S6 Fig SNP-density for chromosomes 1 to 28 in *G*. *g*. *domesticus*. Red bars stand for density of SNPs in genic regions, blue bars stand for SNP-density in non-genic regions.
(TIFF)

S7 Fig Distribution of minor allele frequencies in *G*. *g*. *domesticus* across the whole genome, in genic and in inter-gene regions, respectively. (PNG)

S8 Fig Distribution of pair-wise distances of SNP pairs in *A*. *thaliana*, *H*. *sapiens* and *G*. *g*. *domesticus*. The black vertical line refers to threshold cutting off the upper 1% of data points.
(TIFF)

S9 Fig Distribution of pair-wise *r* ^2^. Distributions of squared correlations *r* ^2^ in *A*. *thaliana* (upper panel), *H*. *sapiens* (central panel), and *G*. *g*. *domesticus* (lower panel) in gene (red) and non-genic (blue) regions.
(TIFF)

S10 Fig Chromosome-wise haplotype diversity in genic and non-genic regions across species. Chromosome-wise haplotype diversity in *G* (red) and *IG* (blue).
(TIF)

S11 Fig Medians of *r* ^2^ in genic and non-genic regions vs. chromosome size in *A*. *thaliana*, *H*. *sapiens*, and *G*. *g*. *domesticus*. Slope of all regression lines does not differ significantly from zero.
(TIF)

S12 FigRelationship between magnitude of LD and the size of regions measured in number of SNPs, across chromosomes in chicken.Genic regions are drawn in red and non-genic regions in blue, X-axis reflects number of SNPs per region, Y-Axis reflects medians of *r* ^2^ (upper panel) or medians of $r_{S}^{2}$ (lower panel). The slope of the linear regression and its corresponding p-value are drown in each panel.(TIF)

S13 FigG/IG differences in medians of *r* ^2^ (upper panel) or medians of $r_{S}^{2}$ (lower panel), against the size of regions (in number of SNPs) across chromosomes in chicken.(TIF)

S1 File Description and content of S2 and S3 Files. (TXT)

S2 File Genotype data for all chromosomes. (ZIP)

S3 File Marker information, containing number of chromosome, relative physical position and affiliation to genic region for all chromosomes. (ZIP)

S1 Table Chromosome-wise averaged medians of pair-wise *r* ^2^, calculated in each *G*, *IG* or *IG’* region for chromosome 1 to 5 in *A*. *thaliana*. D*ifference abs* is the absolute deviation of median in *IG* from median in *G* (or median in *IG’* from median in *IG*) in corresponding regions, *Difference %* gives the percentage of deviation. *p-Val* is the p-value based on Wilcoxon signed rank test. Significant differences (p < 0.05) are marked in red.
(DOCX)

S2 Table Chromosome-wise averaged medians of pair-wise *r* ^2^, calculated in each *G*, *IG* or *IG’* region for chromosome 1 to 22 in *H*. *sapiens*. D*ifference abs* is the absolute deviation of median in *IG* from median in *G* (or median in *IG’* from median in *IG*) in corresponding regions, *Difference %* gives the percentage of deviation. *p-Val* is the p-value based on Wilcoxon signed rank test. Significant differences (p < 0.05) are marked in red.
(DOCX)

S3 Table Chromosome-wise averaged medians of pair-wise *r* ^2^, calculated in each *G*, *IG* or *IG’* region for chromosome 1 to 26 in *G*. *g*. *domesticus*. D*ifference abs* is the absolute deviation of median in *IG* from median in *G* (or median in *IG’* from median in *IG*) in corresponding regions, *Difference %* gives the percentage of deviation. *p-Val* is the p-value based on Wilcoxon signed rank test. Significant differences (p < 0.05) are marked in red.
(DOCX)

S4 Table Chromosome-wise averaged medians of pair-wise *r* ^2^, calculated in each *G*, *IG* or *IG’* region for chromosome 1 to 5 in *A*. *thaliana*. D*ifference abs* is the absolute deviation of median in *IG* from median in *G* (or median in *IG’* from median in *IG*) in corresponding regions, *Difference %* gives the percentage of deviation. *p-Val* is the p-value based on Wilcoxon signed rank test. Significant differences (p < 0.05) are marked in red.
(DOCX)

S5 Table Chromosome-wise averaged medians of pair-wise *r* ^2^, calculated in each *G*, *IG* or *IG’* region for chromosome 1 to 22 in *H*. *sapiens*. D*ifference abs* is the absolute deviation of median in *IG* from median in *G* (or median in *IG’* from median in *IG*) in corresponding regions, *Difference %* gives the percentage of deviation. *p-Val* is the p-value based on Wilcoxon signed rank test. Significant differences (p < 0.05) are marked in red.
(DOCX)

S6 Table Chromosome-wise averaged medians of pair-wise *r* ^2^, calculated in each *G*, *IG* or *IG’* region for chromosome 1 to 26 in *G*. *g*. *domesticus*. D*ifference abs* is the absolute deviation of median in *IG* from median in *G* (or median in *IG’* from median in *IG*) in corresponding regions, *Difference %* gives the percentage of deviation. *p-Val* is the p-value based on Wilcoxon signed rank test. Significant differences (p < 0.05) are marked in red.
(DOCX)

S7 Table Chromosome-wise averaged means of pair-wise *r* ^2^, calculated in each *G*, *IG* or *IG’* region for chromosome 1 to 5 in *A*. *thaliana*. D*ifference abs* is the absolute deviation of mean in *IG* from mean in *G* (or mean in *IG’* from mean in *IG*) in corresponding regions, *Difference %* gives the percentage of deviation. *p-Val* is the p-value based on Wilcoxon signed rank test. Significant differences (p < 0.05) are marked in red.
(DOCX)

S8 Table Chromosome-wise averaged means of pair-wise *r* ^2^, calculated in each *G*, *IG* or *IG’* region for chromosome 1 to 22 in *H*. *sapiens*. D*ifference abs* is the absolute deviation of mean in *IG* from mean in *G* (or mean in *IG’* from mean in *IG*) in corresponding regions, *Difference %* gives the percentage of deviation. *p-Val* is the p-value based on Wilcoxon signed rank test. Significant differences (p < 0.05) are marked in red.
(DOCX)

S9 Table Chromosome-wise averaged means of pair-wise *r* ^2^, calculated in each *G*, *IG* or *IG’* region for chromosome 1 to 26 in *G*. *g*. *domesticus*. D*ifference abs* is the absolute deviation of mean in *IG* from mean in *G* (or mean in *IG’* from mean in *IG*) in corresponding regions, *Difference %* gives the percentage of deviation. *p-Val* is the p-value based on Wilcoxon signed rank test. Significant differences (p < 0.05) are marked in red.
(DOCX)

S10 Table Chromosome-wise averaged haplotype diversity, calculated in each *G*, *IG* or *IG’* region for chromosome 1 to 5 in *A*. *thaliana*. D*ifference abs* is the absolute deviation of mean in *IG* from mean in *G* (or mean in *IG’* from mean in *IG*) in corresponding regions, *Difference %* gives the percentage of deviation. *p-Val* is the p-value based on Wilcoxon signed rank test. Significant differences (p < 0.05) are marked in red.
(DOCX)

S11 Table Chromosome-wise averaged haplotype diversity, calculated in each *G*, *IG* or *IG’* region for chromosome 1 to 22 in *H*. *sapiens*. D*ifference abs* is the absolute deviation of mean in *IG* from mean in *G* (or mean in *IG’* from mean in *IG*) in corresponding regions, *Difference %* gives the percentage of deviation. *p-Val* is the p-value based on Wilcoxon signed rank test. Significant differences (p < 0.05) are marked in red.
(DOCX)

S12 Table Chromosome-wise averaged haplotype diversity, calculated in each *G*, *IG* or *IG’* region for chromosome 1 to 26 in *G*. *g*. *domesticus*. D*ifference abs* is the absolute deviation of mean in *IG* from mean in *G* (or mean in *IG’* from mean in *IG*) in corresponding regions, *Difference %* gives the percentage of deviation. *p-Val* is the p-value based on Wilcoxon signed rank test. Significant differences (p < 0.05) are marked in red.
(DOCX)

S13 TableSlopes and in regressions of chromosome-wise averaged *r* ^2^ and $r_{S}^{2}$ medians on size of the chromosomes.(DOCX)
