# Supplementary figures and images for: A Scale-Corrected Comparison of Linkage Disequilibrium Levels between Genic and Non-Genic Regions
Source: PLoS One. 2015 Oct 30;10(10):e0141216. doi: 10.1371/journal.pone.0141216 (PMC4627745; doi:10.1371/journal.pone.0141216)

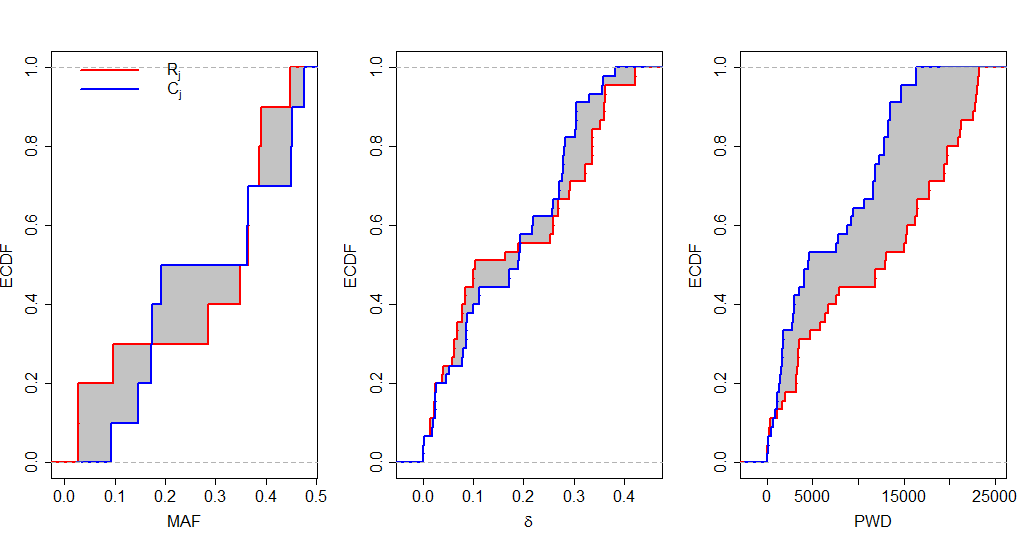

Supplement: S1 Fig — ECDFs for reference set (red) and for a candidate subset (blue), the AMAF(jk) (left), Aδ(jk)(center), and APWD(jk)(right) are marked in grey. (TIFF) [file pone.0141216.s001.tiff]

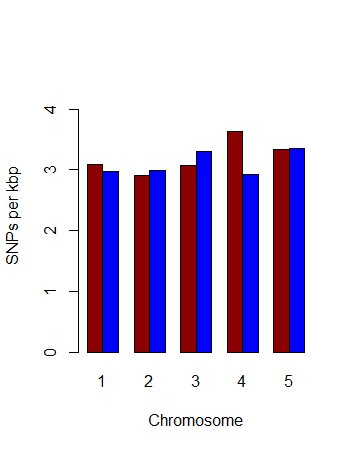

Supplement: S2 Fig — Red bars stand for density of SNPs in genic regions, blue bars stand for SNP-density in non-genic regions. (TIFF) [file pone.0141216.s002.tiff]

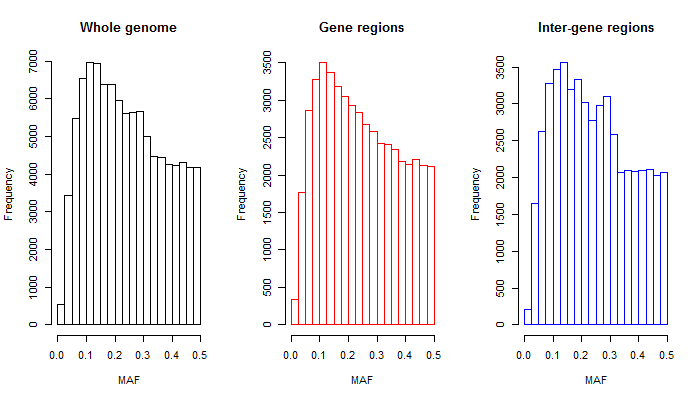

Supplement: S3 Fig — (TIFF) [file pone.0141216.s003.tiff]

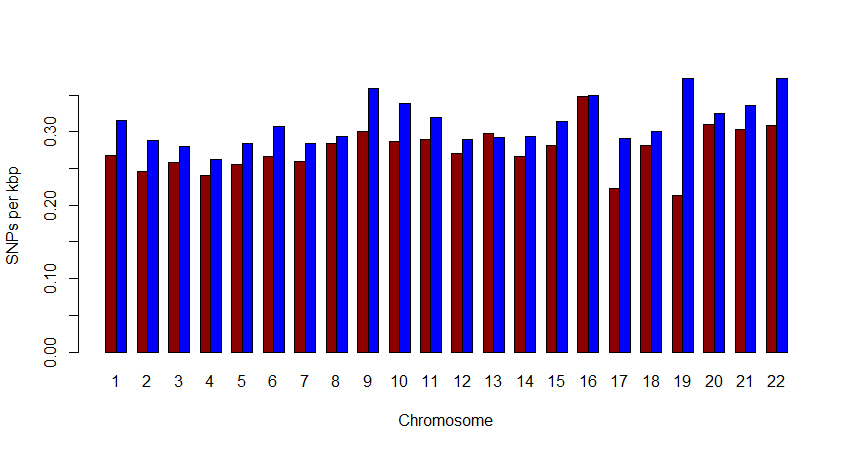

Supplement: S4 Fig — Red bars stand for density of SNPs in genic regions, blue bars stand for SNP-density in non-genic regions. (TIFF) [file pone.0141216.s004.tiff]

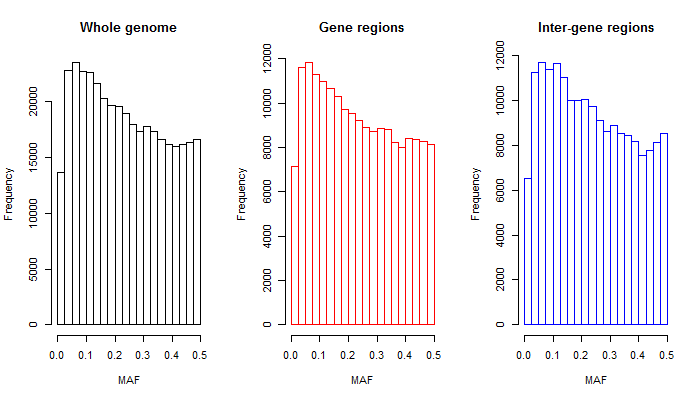

Supplement: S5 Fig — (TIFF) [file pone.0141216.s005.tiff]

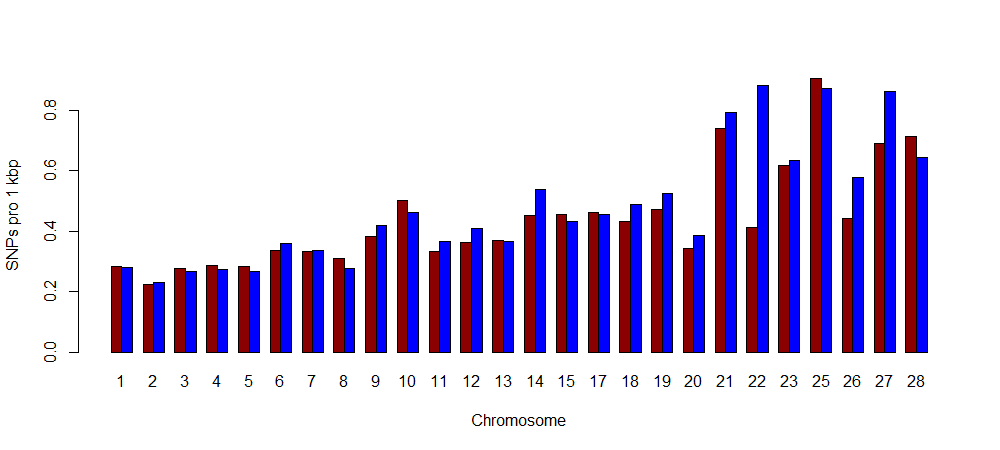

Supplement: S6 Fig — Red bars stand for density of SNPs in genic regions, blue bars stand for SNP-density in non-genic regions. (TIFF) [file pone.0141216.s006.tiff]

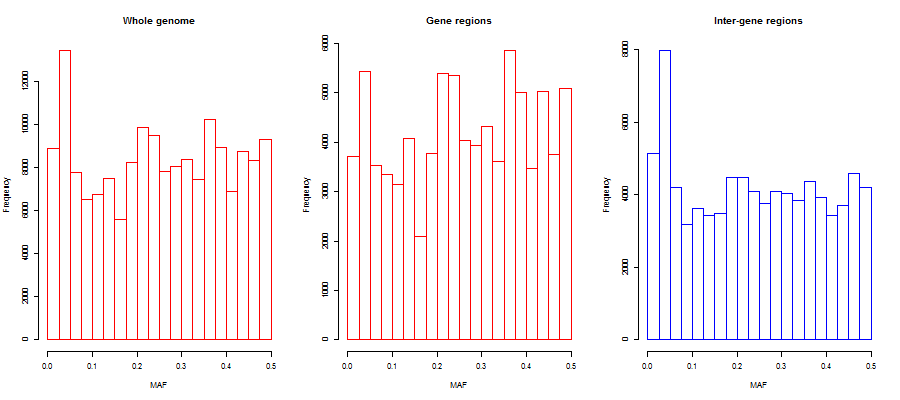

Supplement: S7 Fig — (PNG) [file pone.0141216.s007.png]

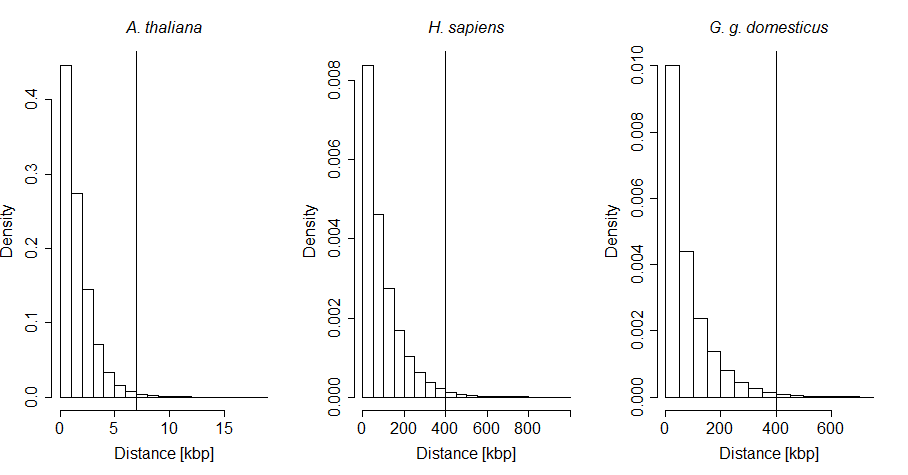

Supplement: S8 Fig — The black vertical line refers to threshold cutting off the upper 1% of data points. (TIFF) [file pone.0141216.s008.tiff]

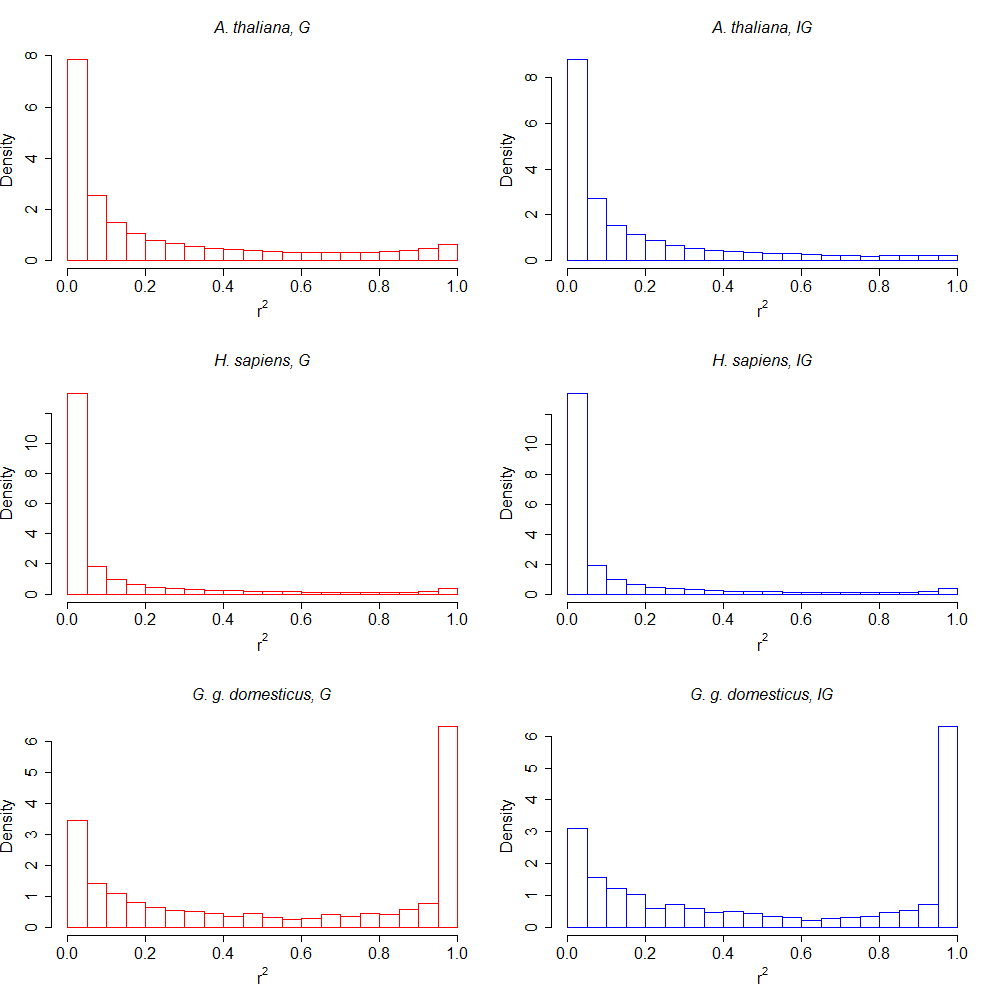

Supplement: S9 Fig — Distributions of squared correlations r 2 in A. thaliana (upper panel), H. sapiens (central panel), and G. g. domesticus (lower panel) in gene (red) and non-genic (blue) regions. (TIFF) [file pone.0141216.s009.tiff]

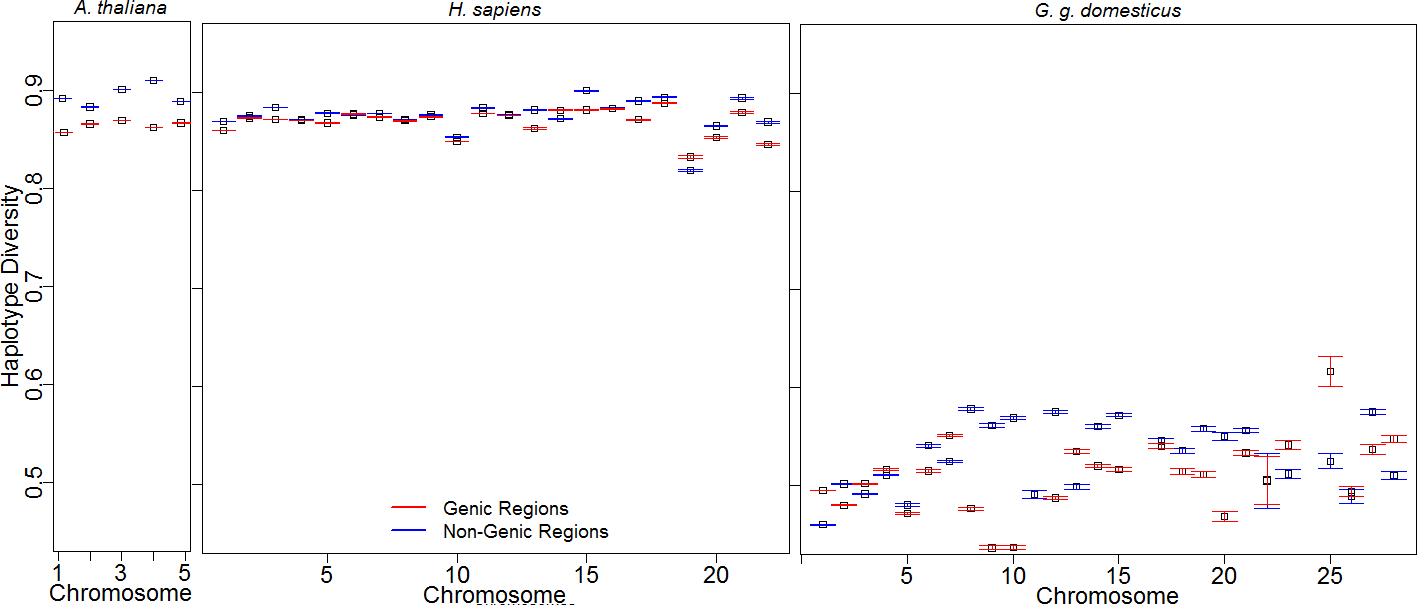

Supplement: S10 Fig — Chromosome-wise haplotype diversity in G (red) and IG (blue). (TIF) [file pone.0141216.s010.tif]

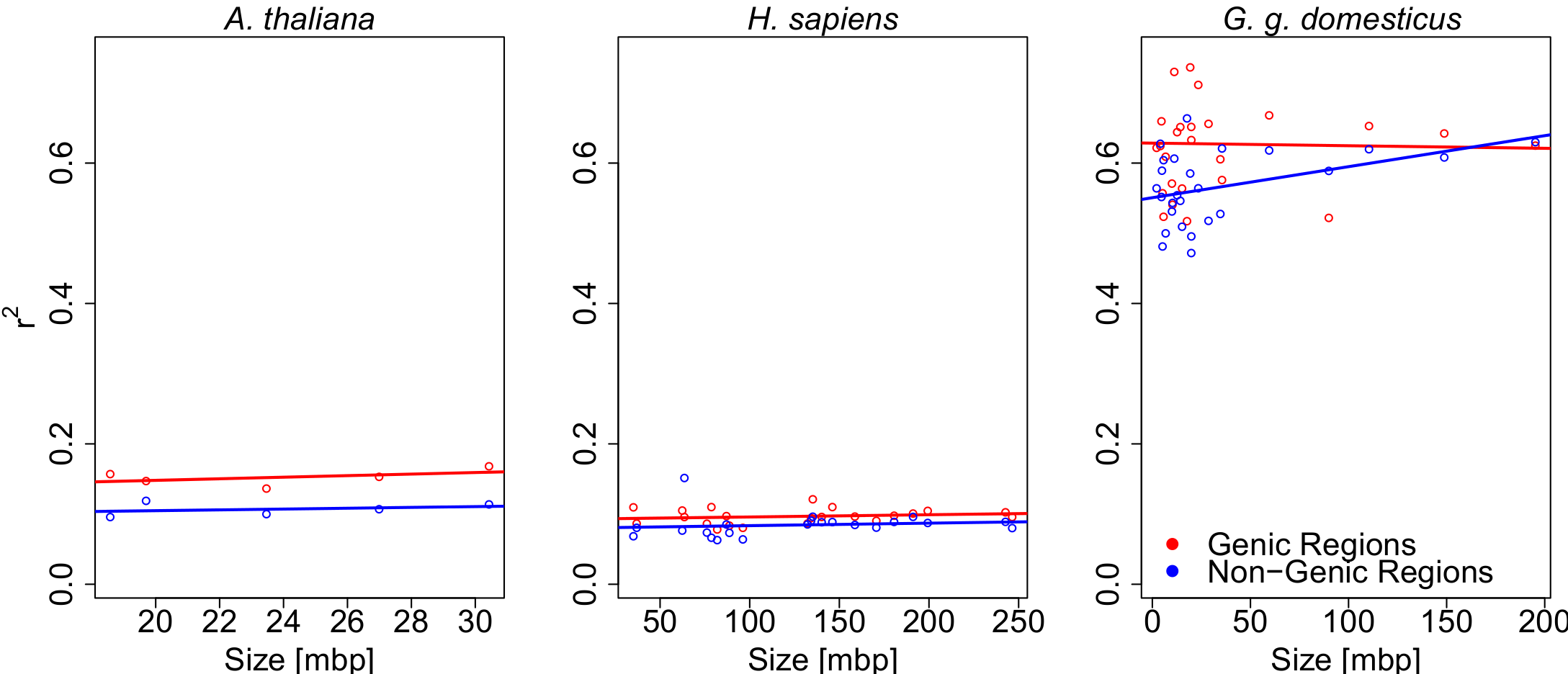

Supplement: S11 Fig — Slope of all regression lines does not differ significantly from zero. (TIF) [file pone.0141216.s011.tif]

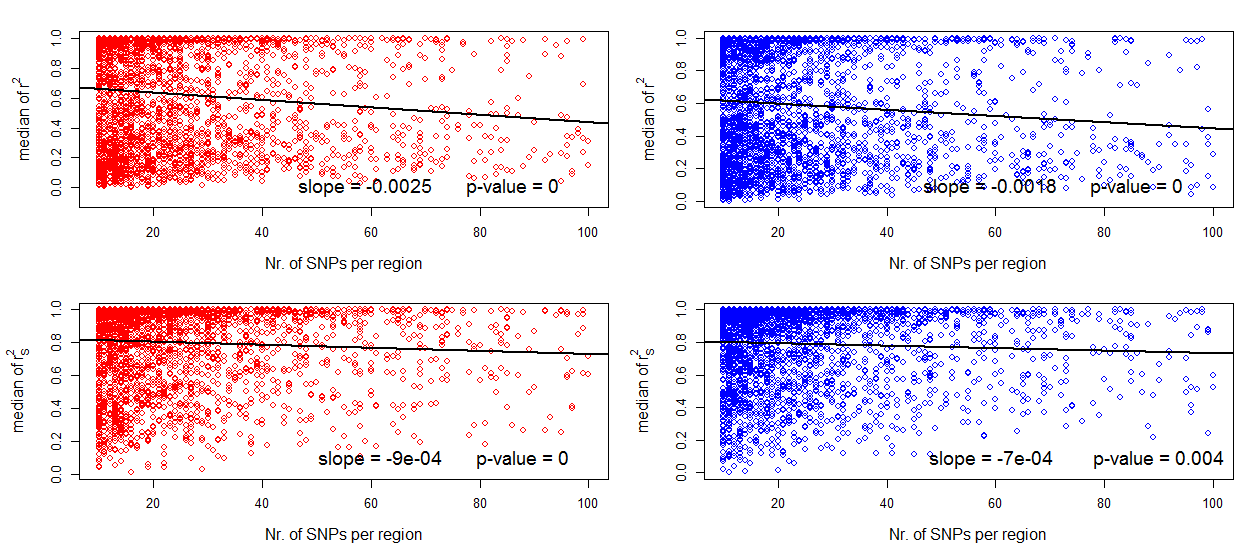

Supplement: S12 Fig — Genic regions are drawn in red and non-genic regions in blue, X-axis reflects number of SNPs per region, Y-Axis reflects medians of r 2 (upper panel) or medians of rS2 (lower panel). The slope of the linear regression and its corresponding p-value are drown in each panel. (TIF) [file pone.0141216.s012.tif]

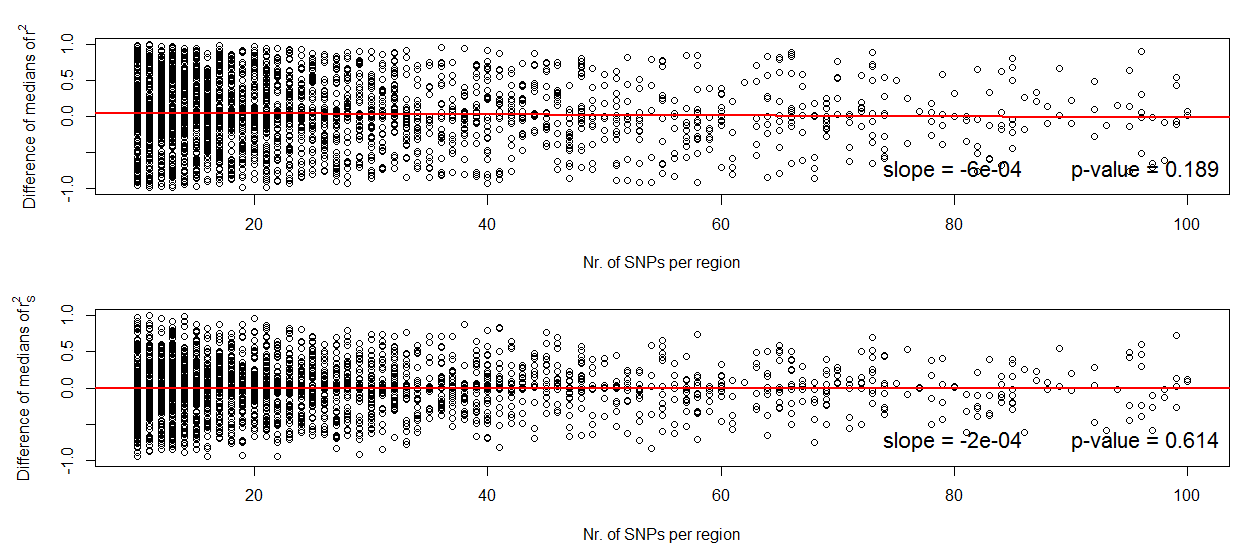

Supplement: S13 Fig — (TIF) [file pone.0141216.s013.tif]
