# Supplementary material for: A Scale-Corrected Comparison of Linkage Disequilibrium Levels between Genic and Non-Genic Regions
Source: PLoS One. 2015 Oct 30;10(10):e0141216. doi: 10.1371/journal.pone.0141216 (PMC4627745; doi:10.1371/journal.pone.0141216)
Supplement: S1 Table — Difference abs is the absolute deviation of median in IG from median in G (or median in IG’ from median in IG) in corresponding regions, Difference % gives the percentage of deviation. p-Val is the p-value based on Wilcoxon signed rank test. Significant differences (p < 0.05) are marked in red. (DOCX) [file pone.0141216.s017.docx]

**S1 Table.** **Chromosome-wise averaged medians of pair-wise**$r^{2}$**, calculated in each *G, IG* or *IG’* region for chromosome 1 to 5 in *A.thaliana*.** D*ifference abs* is the absolute deviation of median in *IG* from median in *G* (or median in *IG’* from median in *IG*) in corresponding regions, *Difference %* gives the percentage of deviation. *p-Val* is the p-value based on Wilcoxon signed rank test. Significant differences (p < 0.05) are marked in red.

|  |  | Median | | Difference | | p-Val | Median | | Difference | | p-Val |
| --- | --- | --- | --- | --- | --- | --- | --- | --- | --- | --- | --- |
| chr | #genes | G | IG | abs | % |  | IG | IG‘ | abs | % |  |
| 1 | 858 | 0.167 | 0.111 | 0.055 | 49.7 | 0 | 0.114 | 0.103 | 0.011 | 9.7 | 0.094 |
| 2 | 348 | 0.147 | 0.118 | 0.029 | 24.6 | 0.016 | 0.119 | 0.094 | 0.025 | 21.0 | 0.200 |
| 3 | 695 | 0.136 | 0.100 | 0.035 | 35.4 | 0 | 0.100 | 0.089 | 0.011 | 11.0 | 0.529 |
| 4 | 669 | 0.155 | 0.096 | 0.059 | 61.6 | 0 | 0.096 | 0.092 | 0.003 | 4.2 | 0.746 |
| 5 | 943 | 0.153 | 0.106 | 0.046 | 43.5 | 0 | 0.107 | 0.111 | -0.004 | -3.7 | 0.254 |
| Genome-wide | | 0.154 | 0.106 | 0.048 | 31.2 | 2 $\cdot$ 10^-16^ | 0.106 | 0.099 | 0.007 | 6.6 | 0.2814 |
