# Supplementary material for: A Scale-Corrected Comparison of Linkage Disequilibrium Levels between Genic and Non-Genic Regions
Source: PLoS One. 2015 Oct 30;10(10):e0141216. doi: 10.1371/journal.pone.0141216 (PMC4627745; doi:10.1371/journal.pone.0141216)
Supplement: S2 Table — Difference abs is the absolute deviation of median in IG from median in G (or median in IG’ from median in IG) in corresponding regions, Difference % gives the percentage of deviation. p-Val is the p-value based on Wilcoxon signed rank test. Significant differences (p < 0.05) are marked in red. (DOCX) [file pone.0141216.s018.docx]

**S2 Table.** **Chromosome-wise averaged medians of pair-wise**$r^{2}$**, calculated in each *G, IG* or *IG’* region for chromosome 1 to 22 in *H.sapiens*.** D*ifference abs* is the absolute deviation of median in *IG* from median in *G* (or median in *IG’* from median in *IG*) in corresponding regions, *Difference %* gives the percentage of deviation. *p-Val* is the p-value based on Wilcoxon signed rank test. Significant differences (p < 0.05) are marked in red.

| chr | #genes | Median | | Difference | | p-Val | Median | | Difference | | p-Val |
| --- | --- | --- | --- | --- | --- | --- | --- | --- | --- | --- | --- |
|  |  | G | IG | abs | % |  | IG | IG‘ | abs | % |  |
| 1 | 661 | 0.096 | 0.080 | 0.016 | 16.7 | 0.038 | 0.080 | 0.083 | -0.003 | -3.7 | 0.661 |
| 2 | 571 | 0.103 | 0.089 | 0.014 | 13.6 | 0.037 | 0.089 | 0.089 | 0 | 0.0 | 0.657 |
| 3 | 437 | 0.105 | 0.087 | 0.018 | 17.1 | 0.181 | 0.087 | 0.084 | 0.003 | 3.4 | 0.223 |
| 4 | 410 | 0.101 | 0.096 | 0.005 | 4.9 | 0.372 | 0.096 | 0.092 | 0.004 | 4.2 | 0.195 |
| 5 | 405 | 0.098 | 0.089 | 0.009 | 9.2 | 0.433 | 0.089 | 0.090 | -0.001 | -1.1 | 0.612 |
| 6 | 406 | 0.090 | 0.081 | 0.009 | 10.0 | 0.991 | 0.081 | 0.083 | -0.002 | -2.5 | 0.103 |
| 7 | 318 | 0.096 | 0.085 | 0.011 | 11.4 | 0.888 | 0.085 | 0.085 | 0 | 0.0 | 0.956 |
| 8 | 322 | 0.110 | 0.089 | 0.021 | 19.1 | 0.064 | 0.089 | 0.082 | 0.007 | 7.9 | 0.497 |
| 9 | 298 | 0.096 | 0.088 | 0.008 | 8.3 | 0.471 | 0.088 | 0.090 | -0.002 | -2.3 | 0.996 |
| 10 | 344 | 0.121 | 0.096 | 0.025 | 20.7 | 0.070 | 0.096 | 0.092 | 0.004 | 4.2 | 0.553 |
| 11 | 344 | 0.094 | 0.091 | 0.003 | 3.2 | 0.857 | 0.091 | 0.082 | 0.009 | 9.9 | 0.674 |
| 12 | 395 | 0.086 | 0.085 | 0.001 | 1.2 | 0.930 | 0.085 | 0.075 | 0.010 | 11.8 | 0.192 |
| 13 | 188 | 0.080 | 0.064 | 0.016 | 20.0 | 0.130 | 0.064 | 0.067 | -0.003 | -4.7 | 0.954 |
| 14 | 244 | 0.097 | 0.085 | 0.012 | 12.4 | 0.134 | 0.085 | 0.078 | 0.007 | 8.2 | 0.196 |
| 15 | 226 | 0.078 | 0.063 | 0.015 | 19.2 | 0.125 | 0.063 | 0.057 | 0.006 | 9.5 | 0.372 |
| 16 | 206 | 0.083 | 0.073 | 0.01 | 12.0 | 0.867 | 0.073 | 0.077 | -0.004 | -5.5 | 0.856 |
| 17 | 253 | 0.110 | 0.066 | 0.044 | 40.0 | 0.000 | 0.066 | 0.062 | 0.004 | 6.1 | 0.214 |
| 18 | 178 | 0.086 | 0.074 | 0.012 | 14.0 | 0.468 | 0.074 | 0.075 | -0.001 | -1.4 | 0.511 |
| 19 | 90 | 0.096 | 0.151 | -0.055 | 57.3 | 0.097 | 0.151 | 0.119 | 0.032 | 21.2 | 0.378 |
| 20 | 177 | 0.105 | 0.076 | 0.029 | 27.7 | 0.004 | 0.076 | 0.075 | 0.001 | 1.3 | 0.682 |
| 21 | 89 | 0.086 | 0.080 | 0.006 | 7.0 | 0.584 | 0.080 | 0.088 | -0.008 | -10.0 | 0.743 |
| 22 | 108 | 0.110 | 0.068 | 0.042 | 38.2 | 0.013 | 0.068 | 0.073 | -0.005 | -7.4 | 0.437 |
| Genome-wide | | 0.098 | 0.084 | 0.013 | 13.6 | 2 $\cdot$ 10^-5^ | 0.0844 | 0.0824 | 0.002 | 2.4 | 0.378 |
