# Supplementary material for: A Scale-Corrected Comparison of Linkage Disequilibrium Levels between Genic and Non-Genic Regions
Source: PLoS One. 2015 Oct 30;10(10):e0141216. doi: 10.1371/journal.pone.0141216 (PMC4627745; doi:10.1371/journal.pone.0141216)
Supplement: S3 Table — Difference abs is the absolute deviation of median in IG from median in G (or median in IG’ from median in IG) in corresponding regions, Difference % gives the percentage of deviation. p-Val is the p-value based on Wilcoxon signed rank test. Significant differences (p < 0.05) are marked in red. (DOCX) [file pone.0141216.s019.docx]

**S3 Table.** **Chromosome-wise averaged medians of pair-wise**$r^{2}$**, calculated in each *G, IG* or *IG’* region for chromosome 1 to 26 in *G. g. domesticus*.** D*ifference abs* is the absolute deviation of median in *IG* from median in *G* (or median in *IG’* from median in *IG*) in corresponding regions, *Difference %* gives the percentage of deviation. *p-Val* is the p-value based on Wilcoxon signed rank test. Significant differences (p < 0.05) are marked in red.

|  |  | Median | | Difference | | p-Val | Median | | Difference | | p-Val |
| --- | --- | --- | --- | --- | --- | --- | --- | --- | --- | --- | --- |
| chr | #genes | G | IG | abs | % |  | IG | IG‘ | abs | % |  |
| 1 | 531 | 0.625 | 0.630 | -0.005 | -0.8 | 0.532 | 0.630 | 0.630 | 0 | 0 | 0.711 |
| 2 | 346 | 0.642 | 0.608 | 0.034 | 5.3 | 0.185 | 0.608 | 0.609 | -0.001 | -0.2 | 0.738 |
| 3 | 310 | 0.651 | 0.620 | 0.031 | 4.8 | 0.176 | 0.620 | 0.623 | -0.003 | -0.5 | 0.525 |
| 4 | 255 | 0.522 | 0.589 | -0.067 | -12.8 | 0.010 | 0.589 | 0.565 | 0.024 | 4.1 | 0.293 |
| 5 | 183 | 0.664 | 0.618 | 0.046 | 6.9 | 0.185 | 0.618 | 0.644 | -0.026 | -4.2 | 0.669 |
| 6 | 140 | 0.605 | 0.528 | 0.077 | 12.7 | 0.010 | 0.528 | 0.563 | -0.035 | -6.6 | 0.204 |
| 7 | 141 | 0.576 | 0.621 | -0.045 | -7.8 | 0.195 | 0.621 | 0.574 | 0.047 | 7.6 | 0.082 |
| 8 | 95 | 0.656 | 0.518 | 0.138 | 21.0 | 0.005 | 0.518 | 0.566 | -0.048 | -9.3 | 0.239 |
| 9 | 83 | 0.711 | 0.564 | 0.147 | 20.7 | 0.002 | 0.564 | 0.551 | 0.013 | 2.3 | 0.772 |
| 10 | 110 | 0.633 | 0.496 | 0.137 | 21.6 | 0.003 | 0.496 | 0.511 | -0.015 | -3.0 | 0.827 |
| 11 | 52 | 0.701 | 0.585 | 0.116 | 16.6 | 0.007 | 0.585 | 0.604 | -0.019 | -3.3 | 0.797 |
| 12 | 94 | 0.651 | 0.472 | 0.179 | 27.5 | 0.000 | 0.472 | 0.546 | -0.074 | -15.7 | 0.174 |
| 13 | 72 | 0.517 | 0.664 | -0.147 | -28.4 | 0.022 | 0.664 | 0.722 | -0.058 | -8.7 | 0.350 |
| 14 | 101 | 0.564 | 0.509 | 0.055 | 9.8 | 0.301 | 0.509 | 0.587 | -0.078 | -15.3 | 0.075 |
| 15 | 75 | 0.644 | 0.554 | 0.090 | 14.0 | 0.098 | 0.554 | 0.551 | 0.003 | 0.5 | 0.790 |
| 17 | 68 | 0.541 | 0.543 | -0.002 | -0.4 | 0.815 | 0.543 | 0.554 | -0.011 | -2.0 | 0.502 |
| 18 | 57 | 0.730 | 0.606 | 0.124 | 17.0 | 0.024 | 0.606 | 0.587 | 0.019 | 3.1 | 0.757 |
| 19 | 60 | 0.571 | 0.531 | 0.040 | 7.0 | 0.553 | 0.531 | 0.561 | -0.030 | -5.7 | 0.340 |
| 20 | 39 | 0.651 | 0.546 | 0.105 | 16.1 | 0.324 | 0.546 | 0.492 | 0.054 | 9.9 | 0.831 |
| 21 | 63 | 0.609 | 0.500 | 0.109 | 17.9 | 0.051 | 0.500 | 0.564 | -0.064 | 12.8 | 0.174 |
| 22 | 7 | 0.624 | 0.628 | -0.004 | -0.6 | 1.000 | 0.628 | 0.685 | -0.057 | -9.1 | 1.000 |
| 23 | 39 | 0.524 | 0.604 | -0.080 | -15.3 | 0.277 | 0.604 | 0.562 | 0.042 | 6.9 | 0.438 |
| 25 | 10 | 0.622 | 0.564 | 0.058 | 9.3 | 0.846 | 0.564 | 0.509 | 0.055 | 9.8 | 0.770 |
| 26 | 26 | 0.814 | 0.589 | 0.225 | 27.6 | 0.012 | 0.589 | 0.631 | -0.042 | -7.1 | 0.354 |
| 27 | 36 | 0.557 | 0.481 | 0.076 | 13.6 | 0.346 | 0.481 | 0.373 | 0.108 | 22.5 | 0.058 |
| 28 | 39 | 0.660 | 0.552 | 0.108 | 16.4 | 0.121 | 0.552 | 0.520 | 0.032 | 5.8 | 0.805 |
| Genome-wide | | 0.621 | 0.584 | 0.037 | 6.0 | 0.008 | 0.584 | 0.591 | -0.007 | -1.2 | 0.57 |
