# Supplementary material for: A Scale-Corrected Comparison of Linkage Disequilibrium Levels between Genic and Non-Genic Regions
Source: PLoS One. 2015 Oct 30;10(10):e0141216. doi: 10.1371/journal.pone.0141216 (PMC4627745; doi:10.1371/journal.pone.0141216)
Supplement: S4 Table — Difference abs is the absolute deviation of median in IG from median in G (or median in IG’ from median in IG) in corresponding regions, Difference % gives the percentage of deviation. p-Val is the p-value based on Wilcoxon signed rank test. Significant differences (p < 0.05) are marked in red. (DOCX) [file pone.0141216.s020.docx]

**S4 Table.** **Chromosome-wise averaged medians of pair-wise**$r_{S}^{2}$**, calculated in each *G, IG* or *IG’* region for chromosome 1 to 5 in *A.thaliana*.** D*ifference abs* is the absolute deviation of median in *IG* from median in *G* (or median in *IG’* from median in *IG*) in corresponding regions, *Difference %* gives the percentage of deviation. *p-Val* is the p-value based on Wilcoxon signed rank test. Significant differences (p < 0.05) are marked in red.

|  |  | Median | | Difference | | p-Val | Median | | Difference | | p-Val |
| --- | --- | --- | --- | --- | --- | --- | --- | --- | --- | --- | --- |
| chr | #genes | G | IG | abs | % |  | IG | IG‘ | abs | % |  |
| 1 | 858 | 0.311 | 0.218 | 0.093 | 29.9 | 10^-6^ | 0.218 | 0.201 | 0.017 | 7.8 | 0.017 |
| 2 | 348 | 0.278 | 0.233 | 0.045 | 16.2 | 0.0018 | 0.233 | 0.203 | 0.030 | 12.9 | 0.130 |
| 3 | 695 | 0.275 | 0.194 | 0.081 | 29.5 | 10^-6^ | 0.194 | 0.185 | 0.009 | 4.6 | 0.411 |
| 4 | 669 | 0.296 | 0.195 | 0.101 | 34.1 | 10^-6^ | 0.195 | 0.196 | -0.001 | -0.5 | 0.941 |
| 5 | 943 | 0.290 | 0.221 | 0.069 | 23.8 | 10^-6^ | 0.221 | 0.225 | -0.004 | -1.8 | 0.284 |
| Genome-wide | | 0.292 | 0.211 | 0.081 | 27.7 | 2 $\cdot$ 10^-16^ | 0.211 | 0.203 | 0.008 | 3.7 | 0.1454 |
