# Supplementary material for: A Scale-Corrected Comparison of Linkage Disequilibrium Levels between Genic and Non-Genic Regions
Source: PLoS One. 2015 Oct 30;10(10):e0141216. doi: 10.1371/journal.pone.0141216 (PMC4627745; doi:10.1371/journal.pone.0141216)
Supplement: S5 Table — Difference abs is the absolute deviation of median in IG from median in G (or median in IG’ from median in IG) in corresponding regions, Difference % gives the percentage of deviation. p-Val is the p-value based on Wilcoxon signed rank test. Significant differences (p < 0.05) are marked in red. (DOCX) [file pone.0141216.s021.docx]

**S5 Table.** **Chromosome-wise averaged medians of pair-wise** $r_{S}^{2}$**, calculated in each *G, IG* or *IG’* region for chromosome 1 to 22 in *H.sapiens*.** D*ifference abs* is the absolute deviation of median in *IG* from median in *G* (or median in *IG’* from median in *IG*) in corresponding regions, *Difference %* gives the percentage of deviation. *p-Val* is the p-value based on Wilcoxon signed rank test. Significant differences (p < 0.05) are marked in red.

| chr | #genes | Median | | Difference | | p-Val | Median | | Difference | | p-Val |
| --- | --- | --- | --- | --- | --- | --- | --- | --- | --- | --- | --- |
|  |  | G | IG | Abs | % |  | IG | IG‘ | abs | % |  |
| 1 | 661 | 0.208 | 0.189 | 0.019 | 9.1 | 0.038 | 0.189 | 0.195 | -0.006 | -3.2 | 0.998 |
| 2 | 571 | 0.213 | 0.201 | 0.012 | 5.6 | 0.037 | 0.201 | 0.193 | 0.008 | 3.9 | 0.168 |
| 3 | 437 | 0.217 | 0.198 | 0.019 | 8.8 | 0.181 | 0.198 | 0.190 | 0.008 | 4.0 | 0.406 |
| 4 | 410 | 0.202 | 0.216 | -0.014 | -6.9 | 0.372 | 0.216 | 0.202 | 0.014 | 6.5 | 0.084 |
| 5 | 405 | 0.226 | 0.203 | 0.023 | 10.2 | 0.433 | 0.203 | 0.205 | -0.002 | -1.0 | 0.982 |
| 6 | 406 | 0.200 | 0.193 | 0.007 | 3.5 | 0.991 | 0.193 | 0.201 | -0.008 | -4.2 | 0.136 |
| 7 | 318 | 0.213 | 0.202 | 0.011 | 5.2 | 0.888 | 0.202 | 0.197 | 0.005 | 2.5 | 0.636 |
| 8 | 322 | 0.231 | 0.211 | 0.020 | 8.7 | 0.064 | 0.211 | 0.192 | 0.019 | 9.0 | 0.116 |
| 9 | 298 | 0.214 | 0.205 | 0.009 | 4.2 | 0.471 | 0.205 | 0.208 | -0.003 | -1.5 | 0.880 |
| 10 | 344 | 0.243 | 0.221 | 0.022 | 9.1 | 0.070 | 0.221 | 0.220 | 0.001 | 0.5 | 0.966 |
| 11 | 344 | 0.216 | 0.198 | 0.018 | 8.3 | 0.857 | 0.198 | 0.197 | 0.001 | 0.6 | 0.645 |
| 12 | 395 | 0.196 | 0.195 | 0.001 | 0.5 | 0.930 | 0.195 | 0.189 | 0.006 | 3.1 | 0.830 |
| 13 | 188 | 0.209 | 0.162 | 0.047 | 22.5 | 0.130 | 0.162 | 0.177 | -0.015 | -9.3 | 0.809 |
| 14 | 244 | 0.213 | 0.208 | 0.005 | 2.3 | 0.134 | 0.208 | 0.190 | 0.018 | 8.7 | 0.382 |
| 15 | 226 | 0.179 | 0.150 | 0.029 | 16.2 | 0.125 | 0.150 | 0.137 | 0.013 | 8.7 | 0.272 |
| 16 | 206 | 0.183 | 0.165 | 0.018 | 9.8 | 0.867 | 0.165 | 0.170 | -0.005 | -3.0 | 0.771 |
| 17 | 253 | 0.225 | 0.158 | 0.067 | 29.8 | 0.000 | 0.158 | 0.148 | 0.010 | 6.3 | 0.350 |
| 18 | 178 | 0.182 | 0.169 | 0.013 | 7.1 | 0.468 | 0.169 | 0.169 | 0 | 0 | 0.690 |
| 19 | 90 | 0.232 | 0.276 | -0.044 | -19.0 | 0.097 | 0.276 | 0.265 | 0.011 | 4.0 | 0.872 |
| 20 | 177 | 0.224 | 0.177 | 0.047 | 20.9 | 0.004 | 0.177 | 0.179 | -0.002 | -1.1 | 0.642 |
| 21 | 89 | 0.200 | 0.196 | 0.004 | 2.0 | 0.584 | 0.196 | 0.217 | -0.021 | -10.7 | 0.479 |
| 22 | 108 | 0.237 | 0.166 | 0.071 | 29.9 | 0.013 | 0.166 | 0.187 | -0.021 | -12.7 | 0.260 |
| Genome-wide | | 0.2119 | 0.1949 | 0.017 | 8.0 | 310^-6^ | 0.1949 | 0.1923 | 0.0026 | 1.3 | 0.188 |
