# Supplementary material for: A Scale-Corrected Comparison of Linkage Disequilibrium Levels between Genic and Non-Genic Regions
Source: PLoS One. 2015 Oct 30;10(10):e0141216. doi: 10.1371/journal.pone.0141216 (PMC4627745; doi:10.1371/journal.pone.0141216)
Supplement: S6 Table — Difference abs is the absolute deviation of median in IG from median in G (or median in IG’ from median in IG) in corresponding regions, Difference % gives the percentage of deviation. p-Val is the p-value based on Wilcoxon signed rank test. Significant differences (p < 0.05) are marked in red. (DOCX) [file pone.0141216.s022.docx]

**S6 Table.** **Chromosome-wise averaged medians of pair-wise**$r_{S}^{2}$**, calculated in each *G, IG* or *IG’* region for chromosome 1 to 26 in *G. g. domesticus*.** D*ifference abs* is the absolute deviation of median in *IG* from median in *G* (or median in *IG’* from median in *IG*) in corresponding regions, *Difference %* gives the percentage of deviation. *p-Val* is the p-value based on Wilcoxon signed rank test. Significant differences (p < 0.05) are marked in red.

|  |  | Median | | Difference | | p-Val | Median | | Difference | | p-Val |
| --- | --- | --- | --- | --- | --- | --- | --- | --- | --- | --- | --- |
| chr | #genes | G | IG | abs | % |  | IG | IG‘ | abs | % |  |
| 1 | 531 | 0.794 | 0.821 | -0.027 | -3.4 | 0.075 | 0.821 | 0.817 | 0.004 | 0.5 | 0.987 |
| 2 | 346 | 0.799 | 0.770 | 0.029 | 3.6 | 0.050 | 0.770 | 0.773 | -0.003 | -0.4 | 0.933 |
| 3 | 310 | 0.827 | 0.822 | 0.005 | 0.6 | 0.688 | 0.822 | 0.830 | -0.008 | -1.0 | 0.809 |
| 4 | 255 | 0.723 | 0.808 | -0.085 | -11.8 | 0.000 | 0.808 | 0.783 | 0.025 | 3.1 | 0.241 |
| 5 | 183 | 0.804 | 0.811 | -0.007 | -0.9 | 0.777 | 0.811 | 0.819 | -0.008 | -1.0 | 0.985 |
| 6 | 140 | 0.809 | 0.755 | 0.054 | 6.7 | 0.037 | 0.755 | 0.767 | -0.012 | -1.6 | 0.470 |
| 7 | 141 | 0.771 | 0.821 | -0.050 | -6.5 | 0.056 | 0.821 | 0.801 | 0.020 | 2.4 | 0.498 |
| 8 | 95 | 0.803 | 0.758 | 0.045 | 5.6 | 0.109 | 0.758 | 0.782 | -0.024 | -3.2 | 0.527 |
| 9 | 83 | 0.853 | 0.777 | 0.076 | 8.9 | 0.023 | 0.777 | 0.779 | -0.002 | -0.3 | 0.471 |
| 10 | 110 | 0.791 | 0.726 | 0.065 | 8.2 | 0.022 | 0.726 | 0.740 | -0.014 | -1.9 | 0.457 |
| 11 | 52 | 0.808 | 0.782 | 0.026 | 3.2 | 0.137 | 0.782 | 0.823 | -0.041 | -5.2 | 0.318 |
| 12 | 94 | 0.800 | 0.731 | 0.069 | 8.6 | 0.067 | 0.731 | 0.768 | -0.037 | -5.1 | 0.226 |
| 13 | 72 | 0.745 | 0.852 | -0.107 | -14.4 | 0.015 | 0.852 | 0.879 | -0.027 | -3.2 | 0.148 |
| 14 | 101 | 0.764 | 0.742 | 0.022 | 2.9 | 0.533 | 0.742 | 0.792 | -0.050 | -6.7 | 0.122 |
| 15 | 75 | 0.841 | 0.783 | 0.058 | 6.9 | 0.042 | 0.783 | 0.765 | 0.018 | 2.3 | 0.603 |
| 17 | 68 | 0.768 | 0.774 | -0.006 | -0.8 | 0.724 | 0.774 | 0.777 | -0.003 | -0.4 | 0.949 |
| 18 | 57 | 0.861 | 0.788 | 0.073 | 8.5 | 0.038 | 0.788 | 0.770 | 0.018 | 2.3 | 0.408 |
| 19 | 60 | 0.786 | 0.759 | 0.027 | 3.4 | 0.271 | 0.759 | 0.805 | -0.046 | -6.1 | 0.348 |
| 20 | 39 | 0.800 | 0.776 | 0.024 | 3.0 | 0.572 | 0.776 | 0.702 | 0.074 | 9.5 | 0.225 |
| 21 | 63 | 0.809 | 0.741 | 0.068 | 8.4 | 0.094 | 0.741 | 0.818 | -0.077 | -10.4 | 0.126 |
| 22 | 7 | 0.827 | 0.844 | -0.017 | -2.1 | 0.402 | 0.844 | 0.898 | -0.054 | -6.4 | 1.000 |
| 23 | 39 | 0.718 | 0.792 | -0.074 | -10.3 | 0.225 | 0.792 | 0.761 | 0.031 | 3.9 | 0.380 |
| 25 | 10 | 0.871 | 0.741 | 0.130 | 14.9 | 0.375 | 0.741 | 0.768 | -0.027 | -3.6 | 1.000 |
| 26 | 26 | 0.895 | 0.840 | 0.055 | 6.2 | 0.034 | 0.840 | 0.851 | -0.011 | -1.3 | 0.681 |
| 27 | 36 | 0.776 | 0.758 | 0.018 | 2.3 | 0.883 | 0.758 | 0.686 | 0.072 | 9.5 | 0.046 |
| 28 | 39 | 0.852 | 0.803 | 0.049 | 5.8 | 0.395 | 0.803 | 0.771 | 0.032 | 4.0 | 0.674 |
| Genome-wide | | 0.795 | 0.791 | 0.004 | 0.5 | 0.059 | 0.791 | 0.794 | -0.003 | -0.4 | 0.438 |
