# Supplementary material for: A Scale-Corrected Comparison of Linkage Disequilibrium Levels between Genic and Non-Genic Regions
Source: PLoS One. 2015 Oct 30;10(10):e0141216. doi: 10.1371/journal.pone.0141216 (PMC4627745; doi:10.1371/journal.pone.0141216)
Supplement: S7 Table — Difference abs is the absolute deviation of mean in IG from mean in G (or mean in IG’ from mean in IG) in corresponding regions, Difference % gives the percentage of deviation. p-Val is the p-value based on Wilcoxon signed rank test. Significant differences (p < 0.05) are marked in red. (DOCX) [file pone.0141216.s023.docx]

**S7 Table.** **Chromosome-wise averaged means of pair-wise**$r^{2}$**, calculated in each *G, IG* or *IG’* region for chromosome 1 to 5 in *A.thaliana*.** D*ifference abs* is the absolute deviation of mean in *IG* from mean in *G* (or mean in *IG’* from mean in *IG*) in corresponding regions, *Difference %* gives the percentage of deviation. *p-Val* is the p-value based on Wilcoxon signed rank test. Significant differences (p < 0.05) are marked in red.

| chr | #genes | Mean | | Difference | | p-Val | Mean | | Difference | | p-Val |
| --- | --- | --- | --- | --- | --- | --- | --- | --- | --- | --- | --- |
|  |  | G | IG | abs | % |  | IG | IG’ | abs | % |  |
| 1 | 858 | 0.256 | 0.196 | 0.060 | 23.4 | 10^-6^ | 0.196 | 0.183 | 0.013 | 6.6 | 0.005 |
| 2 | 348 | 0.235 | 0.207 | 0.028 | 11.9 | 0.003 | 0.207 | 0.190 | 0.017 | 8.2 | 0.049 |
| 3 | 695 | 0.231 | 0.179 | 0.052 | 22.5 | 10^-6^ | 0.179 | 0.172 | 0.007 | 3.9 | 0.423 |
| 4 | 669 | 0.240 | 0.166 | 0.074 | 30.8 | 10^-6^ | 0.166 | 0.170 | -0.004 | -2.0 | 0.437 |
| 5 | 943 | 0.243 | 0.195 | 0.048 | 19.8 | 10^-6^ | 0.195 | 0.203 | -0.008 | -4.0 | 0.026 |
| Genome-wide | | 0.242 | 0.188 | 0.054 | 22.3 | 2 $\cdot$ 10^-16^ | 0.188 | 0.185 | 0.003 | 1.6 | 0.339 |
