# Supplementary material for: A Scale-Corrected Comparison of Linkage Disequilibrium Levels between Genic and Non-Genic Regions
Source: PLoS One. 2015 Oct 30;10(10):e0141216. doi: 10.1371/journal.pone.0141216 (PMC4627745; doi:10.1371/journal.pone.0141216)
Supplement: S8 Table — Difference abs is the absolute deviation of mean in IG from mean in G (or mean in IG’ from mean in IG) in corresponding regions, Difference % gives the percentage of deviation. p-Val is the p-value based on Wilcoxon signed rank test. Significant differences (p < 0.05) are marked in red. (DOCX) [file pone.0141216.s024.docx]

**S8 Table.** **Chromosome-wise averaged means of pair-wise**$r^{2}$**, calculated in each *G, IG* or *IG’* region for chromosome 1 to 22 in *H.sapiens*.** D*ifference abs* is the absolute deviation of mean in *IG* from mean in *G* (or mean in *IG’* from mean in *IG*) in corresponding regions, *Difference %* gives the percentage of deviation. *p-Val* is the p-value based on Wilcoxon signed rank test. Significant differences (p < 0.05) are marked in red.

| chr | #genes | Mean | | Difference | | p-Val | Mean | | Difference | | p-Val |
| --- | --- | --- | --- | --- | --- | --- | --- | --- | --- | --- | --- |
|  |  | G | IG | abs | % |  | IG | IG‘ | abs | % |  |
| 1 | 661 | 0.203 | 0.190 | 0.013 | 6.4 | 0.005 | 0.190 | 0.187 | 0.003 | 1.6 | 0.308 |
| 2 | 571 | 0.199 | 0.191 | 0.008 | 4.0 | 0.150 | 0.191 | 0.186 | 0.005 | 2.6 | 0.308 |
| 3 | 437 | 0.203 | 0.187 | 0.016 | 7.9 | 0.017 | 0.187 | 0.181 | 0.006 | 3.2 | 0.366 |
| 4 | 410 | 0.199 | 0.202 | -0.003 | -1.5 | 0.206 | 0.202 | 0.195 | 0.007 | 3.5 | 0.175 |
| 5 | 405 | 0.206 | 0.190 | 0.016 | 7.8 | 0.007 | 0.190 | 0.191 | -0.001 | -0.5 | 0.984 |
| 6 | 406 | 0.188 | 0.186 | 0.002 | 1.1 | 0.646 | 0.186 | 0.192 | -0.006 | 3.2 | 0.144 |
| 7 | 318 | 0.197 | 0.194 | 0.003 | 1.5 | 0.580 | 0.194 | 0.188 | 0.006 | 3.1 | 0.138 |
| 8 | 322 | 0.209 | 0.191 | 0.018 | 8.6 | 0.080 | 0.191 | 0.186 | 0.005 | 2.6 | 0.607 |
| 9 | 298 | 0.198 | 0.192 | 0.006 | 3.0 | 0.765 | 0.192 | 0.191 | 0.001 | 0.5 | 0.534 |
| 10 | 344 | 0.217 | 0.203 | 0.014 | 6.5 | 0.235 | 0.203 | 0.202 | 0.001 | 0.5 | 0.675 |
| 11 | 344 | 0.201 | 0.193 | 0.008 | 3.9 | 0.393 | 0.193 | 0.189 | 0.004 | 2.1 | 0.564 |
| 12 | 395 | 0.191 | 0.187 | 0.004 | 2.1 | 0.328 | 0.187 | 0.181 | 0.006 | 3.2 | 0.517 |
| 13 | 188 | 0.193 | 0.169 | 0.024 | 12.4 | 0.001 | 0.169 | 0.175 | -0.006 | -3.6 | 0.953 |
| 14 | 244 | 0.192 | 0.188 | 0.004 | 2.1 | 0.374 | 0.188 | 0.181 | 0.007 | 3.7 | 0.277 |
| 15 | 226 | 0.179 | 0.163 | 0.016 | 8.9 | 0.128 | 0.163 | 0.153 | 0.010 | 6.1 | 0.051 |
| 16 | 206 | 0.185 | 0.176 | 0.009 | 4.9 | 0.406 | 0.176 | 0.171 | 0.005 | 2.8 | 0.373 |
| 17 | 253 | 0.204 | 0.166 | 0.038 | 18.6 | 0.000 | 0.166 | 0.158 | 0.008 | 4.8 | 0.136 |
| 18 | 178 | 0.175 | 0.174 | 0.001 | 0.6 | 0.975 | 0.174 | 0.175 | -0.001 | -0.6 | 0.670 |
| 19 | 90 | 0.206 | 0.230 | -0.024 | -11.7 | 0.351 | 0.230 | 0.223 | 0.007 | 3.0 | 0.636 |
| 20 | 177 | 0.210 | 0.191 | 0.019 | 9.1 | 0.050 | 0.191 | 0.183 | 0.008 | 4.2 | 0.547 |
| 21 | 89 | 0.195 | 0.188 | 0.007 | 3.6 | 0.740 | 0.188 | 0.188 | 0.000 | 0.0 | 0.825 |
| 22 | 108 | 0.212 | 0.173 | 0.039 | 18.4 | 0.006 | 0.173 | 0.178 | -0.005 | -2.9 | 0.392 |
| Genome-wide | | 0.199 | 0.188 | 0.011 | 5.3 | 6 $\cdot$ 10^-8^ | 0.188 | 0.185 | 0.004 | 1.9 | 0.012 |
