# Supplementary material for: A Scale-Corrected Comparison of Linkage Disequilibrium Levels between Genic and Non-Genic Regions
Source: PLoS One. 2015 Oct 30;10(10):e0141216. doi: 10.1371/journal.pone.0141216 (PMC4627745; doi:10.1371/journal.pone.0141216)
Supplement: S9 Table — Difference abs is the absolute deviation of mean in IG from mean in G (or mean in IG’ from mean in IG) in corresponding regions, Difference % gives the percentage of deviation. p-Val is the p-value based on Wilcoxon signed rank test. Significant differences (p < 0.05) are marked in red. (DOCX) [file pone.0141216.s025.docx]

**S9 Table.** **Chromosome-wise averaged means of pair-wise**$r^{2}$**, calculated in each *G, IG* or *IG’* region for chromosome 1 to 26 in *G. g. domesticus*.** D*ifference abs* is the absolute deviation of mean in *IG* from mean in *G* (or mean in *IG’* from mean in *IG*) in corresponding regions, *Difference %* gives the percentage of deviation. *p-Val* is the p-value based on Wilcoxon signed rank test. Significant differences (p < 0.05) are marked in red.

| chr | #genes | Mean | | Difference | | p-Val | Mean | | Difference | | p-Val |
| --- | --- | --- | --- | --- | --- | --- | --- | --- | --- | --- | --- |
|  |  | G | IG | abs | % |  | IG | IG‘ | abs | % |  |
| 1 | 531 | 0.645 | 0.644 | 0.001 | 0.2 | 0.850 | 0.644 | 0.643 | 0.001 | 0.2 | 0.891 |
| 2 | 346 | 0.648 | 0.622 | 0.026 | 4.0 | 0.046 | 0.622 | 0.627 | -0.005 | -0.8 | 0.615 |
| 3 | 310 | 0.668 | 0.625 | 0.043 | 6.4 | 0.022 | 0.625 | 0.637 | -0.012 | -1.9 | 0.170 |
| 4 | 255 | 0.559 | 0.602 | -0.040 | -7.7 | 0.013 | 0.602 | 0.586 | 0.016 | 2.7 | 0.177 |
| 5 | 183 | 0.678 | 0.626 | 0.052 | 7.7 | 0.031 | 0.626 | 0.661 | -0.035 | -5.6 | 0.011 |
| 6 | 140 | 0.629 | 0.593 | 0.036 | 5.7 | 0.095 | 0.593 | 0.600 | -0.007 | -1.2 | 0.542 |
| 7 | 141 | 0.615 | 0.632 | -0.020 | -2.7 | 0.381 | 0.632 | 0.617 | 0.015 | 2.43 | 0.322 |
| 8 | 95 | 0.687 | 0.570 | 0.117 | 17.0 | 0.000 | 0.570 | 0.568 | 0.002 | 0.4 | 0.825 |
| 9 | 83 | 0.669 | 0.596 | 0.073 | 10.9 | 0.012 | 0.596 | 0.599 | -0.003 | -0.5 | 0.958 |
| 10 | 110 | 0.660 | 0.545 | 0.115 | 17.4 | 0.000 | 0.545 | 0.550 | -0.005 | -0.9 | 0.732 |
| 11 | 52 | 0.709 | 0.595 | 0.114 | 16.1 | 0.001 | 0.595 | 0.601 | -0.006 | -1.0 | 0.788 |
| 12 | 94 | 0.677 | 0.552 | 0.125 | 18.5 | 0.000 | 0.552 | 0.572 | -0.020 | -3.6 | 0.205 |
| 13 | 72 | 0.563 | 0.660 | -0.100 | -17.2 | 0.011 | 0.660 | 0.686 | -0.026 | -3.9 | 0.130 |
| 14 | 101 | 0.609 | 0.569 | 0.040 | 6.6 | 0.227 | 0.569 | 0.604 | -0.035 | -6.2 | 0.015 |
| 15 | 75 | 0.658 | 0.581 | 0.077 | 11.7 | 0.049 | 0.581 | 0.576 | 0.005 | 0.9 | 0.835 |
| 17 | 68 | 0.598 | 0.590 | 0.008 | 1.4 | 0.939 | 0.590 | 0.590 | 0 | 0 | 0.959 |
| 18 | 57 | 0.719 | 0.631 | 0.088 | 12.2 | 0.013 | 0.631 | 0.615 | 0.016 | 2.5 | 0.328 |
| 19 | 60 | 0.598 | 0.581 | 0.017 | 2.8 | 0.800 | 0.581 | 0.6 | -0.019 | -3.3 | 0.473 |
| 20 | 39 | 0.686 | 0.602 | 0.084 | 12.2 | 0.171 | 0.602 | 0.567 | 0.035 | 5.8 | 0.117 |
| 21 | 63 | 0.639 | 0.554 | 0.085 | 13.3 | 0.040 | 0.554 | 0.562 | -0.008 | -1.4 | 0.649 |
| 22 | 7 | 0.619 | 0.65 | -0.030 | -5.0 | 0.578 | 0.650 | 0.653 | -0.003 | -0.5 | 0.578 |
| 23 | 39 | 0.582 | 0.624 | -0.040 | -7.2 | 0.435 | 0.624 | 0.577 | 0.047 | 7.5 | 0.019 |
| 25 | 10 | 0.616 | 0.543 | 0.073 | 11.9 | 0.557 | 0.543 | 0.560 | -0.017 | -3.1 | 1.000 |
| 26 | 26 | 0.810 | 0.613 | 0.197 | 24.3 | 0.002 | 0.613 | 0.632 | -0.019 | -3.1 | 0.745 |
| 27 | 36 | 0.567 | 0.511 | 0.056 | 9.9 | 0.279 | 0.511 | 0.476 | 0.035 | 6.9 | 0.131 |
| 28 | 39 | 0.679 | 0.57 | 0.109 | 16.1 | 0.036 | 0.570 | 0.560 | 0.010 | 1.8 | 0.664 |
| Genome-wide | | 0.642 | 0.609 | 0.033 | 5.2 | 8 $\cdot$ 10^-7^ | 0.6091 | 0.6124 | -0.003 | -0.5 | 0.290 |
