# Supplementary material for: A Scale-Corrected Comparison of Linkage Disequilibrium Levels between Genic and Non-Genic Regions
Source: PLoS One. 2015 Oct 30;10(10):e0141216. doi: 10.1371/journal.pone.0141216 (PMC4627745; doi:10.1371/journal.pone.0141216)
Supplement: S10 Table — Difference abs is the absolute deviation of mean in IG from mean in G (or mean in IG’ from mean in IG) in corresponding regions, Difference % gives the percentage of deviation. p-Val is the p-value based on Wilcoxon signed rank test. Significant differences (p < 0.05) are marked in red. (DOCX) [file pone.0141216.s026.docx]

**S10 Table.** **Chromosome-wise averaged haplotype diversity, calculated in each *G, IG* or *IG’* region for chromosome 1 to 5 in *A.thaliana*.** D*ifference abs* is the absolute deviation of mean in *IG* from mean in *G* (or mean in *IG’* from mean in *IG*) in corresponding regions, *Difference %* gives the percentage of deviation. *p-Val* is the p-value based on Wilcoxon signed rank test. Significant differences (p < 0.05) are marked in red.

| chr | #genes | Mean | | Difference | | p-Val | Mean | | Difference | | p-Val |
| --- | --- | --- | --- | --- | --- | --- | --- | --- | --- | --- | --- |
|  |  | G | IG | abs | % |  | IG | IG’ | abs | % |  |
| 1 | 858 | 0.857 | 0.892 | -0.034 | -3.8 | 0 | 0.892 | 0.898 | -0.006 | -0.7 | 0.012 |
| 2 | 348 | 0.865 | 0.883 | -0.018 | -2.0 | 0.007 | 0.883 | 0.891 | -0.008 | -0.9 | 0.083 |
| 3 | 695 | 0.869 | 0.901 | -0.031 | -3.5 | 0 | 0.901 | 0.901 | 0 | -0.1 | 0.832 |
| 4 | 669 | 0.862 | 0.910 | -0.048 | -5.3 | 0 | 0.910 | 0.904 | 0.006 | 0.6 | 0.049 |
| 5 | 943 | 0.866 | 0.889 | -0.023 | -2.6 | 0 | 0.889 | 0.886 | 0.003 | 0.4 | 0.268 |
| Genome-wide | | 0.864 | 0.895 | -0.032 | -3.5 | 0 | 0.895 | 0.896 | -0.005 | -0.1 | 0.747 |
