# Supplementary material for: A Scale-Corrected Comparison of Linkage Disequilibrium Levels between Genic and Non-Genic Regions
Source: PLoS One. 2015 Oct 30;10(10):e0141216. doi: 10.1371/journal.pone.0141216 (PMC4627745; doi:10.1371/journal.pone.0141216)
Supplement: S11 Table — Difference abs is the absolute deviation of mean in IG from mean in G (or mean in IG’ from mean in IG) in corresponding regions, Difference % gives the percentage of deviation. p-Val is the p-value based on Wilcoxon signed rank test. Significant differences (p < 0.05) are marked in red. (DOCX) [file pone.0141216.s027.docx]

**S11 Table.** **Chromosome-wise averaged haplotype diversity, calculated in each *G, IG* or *IG’* region for chromosome 1 to 22 in *H.sapiens*.** D*ifference abs* is the absolute deviation of mean in *IG* from mean in *G* (or mean in *IG’* from mean in *IG*) in corresponding regions, *Difference %* gives the percentage of deviation. *p-Val* is the p-value based on Wilcoxon signed rank test. Significant differences (p < 0.05) are marked in red.

| chr | #genes | Mean | | Difference | | p-Val | Mean | | Difference | | p-Val |
| --- | --- | --- | --- | --- | --- | --- | --- | --- | --- | --- | --- |
|  |  | G | IG | abs | % |  | IG | IG‘ | abs | % |  |
| 1 | 661 | 0.861 | 0.870 | -0.009 | -1.1 | 0.059 | 0.870 | 0.868 | 0.002 | 0.2 | 0.930 |
| 2 | 571 | 0.874 | 0.876 | -0.002 | -0.2 | 0.867 | 0.876 | 0.879 | -0.004 | -0.4 | 0.358 |
| 3 | 437 | 0.872 | 0.884 | -0.012 | -1.4 | 0.036 | 0.884 | 0.889 | -0.005 | -0.5 | 0.611 |
| 4 | 410 | 0.871 | 0.872 | -0.001 | -0.1 | 0.585 | 0.872 | 0.881 | -0.009 | -0.9 | 0.112 |
| 5 | 405 | 0.868 | 0.878 | -0.010 | -1.1 | 0.122 | 0.878 | 0.872 | 0.007 | 0.8 | 0.304 |
| 6 | 406 | 0.878 | 0.877 | 0.001 | 0.1 | 0.567 | 0.877 | 0.873 | 0.004 | 0.5 | 0.311 |
| 7 | 318 | 0.874 | 0.878 | -0.004 | -0.5 | 0.577 | 0.878 | 0.880 | -0.002 | -0.2 | 0.283 |
| 8 | 322 | 0.871 | 0.872 | -0.001 | -0.1 | 0.980 | 0.872 | 0.883 | -0.011 | -1.2 | 0.136 |
| 9 | 298 | 0.875 | 0.877 | -0.002 | -0.2 | 0.464 | 0.877 | 0.881 | -0.005 | -0.5 | 0.550 |
| 10 | 344 | 0.850 | 0.854 | -0.004 | -0.5 | 0.778 | 0.854 | 0.856 | -0.002 | -0.2 | 0.677 |
| 11 | 344 | 0.878 | 0.884 | -0.005 | -0.6 | 0.325 | 0.884 | 0.876 | 0.008 | 0.9 | 0.347 |
| 12 | 395 | 0.876 | 0.877 | -0.001 | -0.1 | 0.503 | 0.877 | 0.881 | -0.004 | -0.4 | 0.519 |
| 13 | 188 | 0.863 | 0.882 | -0.019 | -2.2 | 0.013 | 0.882 | 0.874 | 0.008 | 0.9 | 0.839 |
| 14 | 244 | 0.881 | 0.873 | 0.009 | 1.0 | 0.021 | 0.873 | 0.879 | -0.006 | -0.7 | 0.432 |
| 15 | 226 | 0.882 | 0.902 | -0.020 | -2.2 | 0.006 | 0.902 | 0.903 | -0.002 | -0.2 | 0.676 |
| 16 | 206 | 0.883 | 0.883 | 0 | -0.1 | 0.760 | 0.883 | 0.891 | -0.007 | -0.8 | 0.531 |
| 17 | 253 | 0.872 | 0.891 | -0.019 | -2.1 | 0.003 | 0.891 | 0.898 | -0.007 | -0.8 | 0.378 |
| 18 | 178 | 0.889 | 0.895 | -0.006 | -0.7 | 0.940 | 0.895 | 0.893 | 0.002 | 0.2 | 0.474 |
| 19 | 90 | 0.834 | 0.820 | 0.014 | 1.7 | 0.412 | 0.820 | 0.825 | -0.005 | -0.6 | 0.906 |
| 20 | 177 | 0.854 | 0.865 | -0.012 | -1.4 | 0.100 | 0.865 | 0.873 | -0.008 | -0.9 | 0.756 |
| 21 | 89 | 0.879 | 0.894 | -0.015 | -1.7 | 0.171 | 0.894 | 0.882 | 0.012 | 1.3 | 0.338 |
| 22 | 108 | 0.847 | 0.869 | -0.023 | -2.6 | 0.007 | 0.869 | 0.859 | 0.01 | 1.2 | 0.398 |
| Genome-wide | | 0.871 | 0.876 | -0.006 | -0.7 | 0.001 | 0.876 | 0.878 | -0.001 | -0.2 | 0.264 |
