# Supplementary material for: A Scale-Corrected Comparison of Linkage Disequilibrium Levels between Genic and Non-Genic Regions
Source: PLoS One. 2015 Oct 30;10(10):e0141216. doi: 10.1371/journal.pone.0141216 (PMC4627745; doi:10.1371/journal.pone.0141216)
Supplement: S12 Table — Difference abs is the absolute deviation of mean in IG from mean in G (or mean in IG’ from mean in IG) in corresponding regions, Difference % gives the percentage of deviation. p-Val is the p-value based on Wilcoxon signed rank test. Significant differences (p < 0.05) are marked in red. (DOCX) [file pone.0141216.s028.docx]

**S12 Table.** **Chromosome-wise averaged haplotype diversity, calculated in each *G, IG* or *IG’* region for chromosome 1 to 26 in *G. g. domesticus*.** D*ifference abs* is the absolute deviation of mean in *IG* from mean in *G* (or mean in *IG’* from mean in *IG*) in corresponding regions, *Difference %* gives the percentage of deviation. *p-Val* is the p-value based on Wilcoxon signed rank test. Significant differences (p < 0.05) are marked in red.

| chr | #genes | Mean | | Difference | | p-Val | Mean | | Difference | | p-Val |
| --- | --- | --- | --- | --- | --- | --- | --- | --- | --- | --- | --- |
|  |  | G | IG | abs | % |  | IG | IG‘ | abs | % |  |
| 1 | 531 | 0.495 | 0.460 | 0.035 | 7.1 | 0.000 | 0.460 | 0.456 | 0.005 | 1.1 | 0.918 |
| 2 | 346 | 0.480 | 0.501 | -0.021 | -4.4 | 0.028 | 0.501 | 0.474 | 0.027 | 5.4 | 0.006 |
| 3 | 308 | 0.502 | 0.491 | 0.011 | 2.2 | 0.573 | 0.491 | 0.470 | 0.022 | 4.5 | 0.002 |
| 4 | 255 | 0.516 | 0.511 | 0.006 | 1.2 | 0.914 | 0.511 | 0.501 | 0.010 | 2.0 | 0.343 |
| 5 | 181 | 0.472 | 0.480 | -0.008 | -1.7 | 0.537 | 0.480 | 0.433 | 0.047 | 9.8 | 0.000 |
| 6 | 140 | 0.515 | 0.541 | -0.026 | -5.0 | 0.014 | 0.541 | 0.509 | 0.032 | 5.9 | 0.034 |
| 7 | 141 | 0.551 | 0.525 | 0.026 | 4.7 | 0.494 | 0.525 | 0.491 | 0.034 | 6.5 | 0.124 |
| 8 | 95 | 0.476 | 0.578 | -0.102 | -21.4 | 0.000 | 0.578 | 0.560 | 0.018 | 3.1 | 0.217 |
| 9 | 83 | 0.436 | 0.561 | -0.125 | -28.7 | 0.000 | 0.561 | 0.529 | 0.032 | 5.7 | 0.128 |
| 10 | 110 | 0.437 | 0.569 | -0.132 | -30.2 | 0.000 | 0.569 | 0.558 | 0.011 | 1.9 | 0.969 |
| 11 | 45 | 0.400 | 0.491 | -0.091 | -22.8 | 0.012 | 0.491 | 0.488 | 0.003 | 0.6 | 0.858 |
| 12 | 94 | 0.487 | 0.575 | -0.088 | -18.1 | 0.000 | 0.575 | 0.563 | 0.012 | 2.1 | 0.371 |
| 13 | 72 | 0.535 | 0.499 | 0.036 | 6.7 | 0.125 | 0.499 | 0.490 | 0.009 | 1.8 | 0.656 |
| 14 | 101 | 0.520 | 0.560 | -0.040 | -7.7 | 0.062 | 0.560 | 0.512 | 0.048 | 8.6 | 0.001 |
| 15 | 75 | 0.516 | 0.572 | -0.055 | -10.7 | 0.013 | 0.572 | 0.571 | 0.000 | 0.0 | 0.851 |
| 17 | 68 | 0.540 | 0.545 | -0.005 | -0.9 | 0.934 | 0.545 | 0.532 | 0.014 | 2.6 | 0.345 |
| 18 | 57 | 0.514 | 0.535 | -0.021 | -4.1 | 0.249 | 0.535 | 0.542 | -0.006 | -1.1 | 0.639 |
| 19 | 60 | 0.511 | 0.558 | -0.047 | -9.2 | 0.121 | 0.558 | 0.540 | 0.018 | 3.2 | 0.420 |
| 20 | 39 | 0.469 | 0.550 | -0.081 | -17.3 | 0.032 | 0.550 | 0.548 | 0.002 | 0.4 | 0.704 |
| 21 | 63 | 0.533 | 0.556 | -0.023 | -4.3 | 0.491 | 0.556 | 0.548 | 0.008 | 1.4 | 0.719 |
| 22 | 7 | 0.506 | 0.505 | 0.001 | 0.2 | 1.000 | 0.505 | 0.532 | -0.028 | -5.5 | 0.578 |
| 23 | 39 | 0.541 | 0.512 | 0.030 | 5.5 | 0.403 | 0.512 | 0.510 | 0.002 | 0.4 | 0.845 |
| 25 | 10 | 0.616 | 0.525 | 0.092 | 14.9 | 0.106 | 0.525 | 0.586 | -0.062 | -11.8 | 0.160 |
| 26 | 26 | 0.494 | 0.489 | 0.005 | 1.0 | 0.980 | 0.489 | 0.500 | -0.011 | -2.2 | 0.269 |
| 27 | 36 | 0.537 | 0.575 | -0.038 | -7.1 | 0.293 | 0.575 | 0.579 | -0.004 | -0.7 | 0.379 |
| 28 | 39 | 0.547 | 0.510 | 0.037 | 6.8 | 0.521 | 0.510 | 0.508 | 0.002 | 0.4 | 0.841 |
| Genome-wide | | 0.499 | 0.513 | -0.015 | -3.0 | 10^-6^ | 0.513 | 0.496 | 0.017 | 3.4 | 0.001 |
