# Supplementary material for: A Scale-Corrected Comparison of Linkage Disequilibrium Levels between Genic and Non-Genic Regions
Source: PLoS One. 2015 Oct 30;10(10):e0141216. doi: 10.1371/journal.pone.0141216 (PMC4627745; doi:10.1371/journal.pone.0141216)
Supplement: S13 Table — (DOCX) [file pone.0141216.s029.docx]

**S13 Table. Slopes and in regressions of chromosome-wise averaged $r^{2}$and $r_{S}^{2}$medians on size of the chromosomes**.

| **Species** |  | Genic regions | | Non-genic regions | |
| --- | --- | --- | --- | --- | --- |
|  |  | slope | p-value | slope | p-value |
| ***A. thaliana*** | $r^{2}$ | 0.00111 | 0.4254 | 0.00058 | 0.6199 |
|  | $r_{S}^{2}$ | 0.00162 | 0.3280 | 0.00074 | 0.7249 |
| ***H. sapiens*** | $r^{2}$ | 0,00003 | 0.4210 | 0.00004 | 0.5870 |
|  | $r_{S}^{2}$ | 0.00001 | 0.9290 | 0.00011 | 0.2980 |
| ***G. g. domesticus*** | $r^{2}$ | -0.00004 | 0.9030 | 0.00044 | 0.0360 |
|  | $r_{S}^{2}$ | -0.00014 | 0.4460 | 0.00019 | 0.2190 |
